# Unraveling the neural basis of repeated moral decisions with mouse tracking and fMRI

**DOI:** 10.1162/IMAG.a.1047

**Published:** 2025-12-16

**Authors:** Xinyi Julia Xu, Guochun Yang, Jiamin Huang, Ruien Wang, Haiyan Wu

**Affiliations:** Centre for Cognitive and Brain Sciences and Department of Psychology, University of Macau, Macau, China; Guangdong Institute of Intelligence Science and Technology, Guangzhou, China

**Keywords:** choice consistency, moral decisions, dishonesty, reward, fMRI, mouse tracking

## Abstract

Tracking response history and current rewards is critical for making moral decisions. By integrating functional magnetic resonance imaging (fMRI) and mouse tracking (MT) with a value-based moral decision task, we quantify the level of choice conflict with the MT metric—area under the curve (AUC), and examine how individuals incorporate information from the response history to make repeated moral decisions. Our study uses response entropy and cumulative responses (CR) to define choice consistency on both the subject level and trial level. We find that a stronger correlation between AUC and response entropy is mediated by the weight of reward in decisions. Regarding the fMRI results, the precuneus tracks AUC and increases across time. Across the whole brain, we compare the pattern of how the human brain encodes AUC with several NeuroSynth templates, and find that neural similarity of reward decreased and was correlated with entropy and the weight of relative reward. Meanwhile, multivariate representations in cognitive control and self-referential brain regions such as inferior frontal gyrus (IFG), anterior cingulate cortex (ACC), and temporoparietal junction (TPJ) encode the weight of relative reward and CR together. Finally, the functional connectivity (FC) analysis show that ACC, as well as the FC between ACC and precuneus, becomes more prominent across time. Through understanding choice conflict and response history, our research sheds light on its significance in multi-trial moral decision from the consistency perspective. These findings establish a foundation for exploring the underlying mechanisms involved in repeated decisions with conflict processes.

## 1 Introduction

In daily life, individuals are likely to confront numerous instances of repetitive or analogous decision-making contexts. While we may adapt our decision-making strategies to the same situation in different contexts (Agranov & Ortoleva, 2017), there is a tracking of consistency that permeates our repeated decisions. Choice consistency refers to repeating the previous choices, which is quantified by cumulative responses (CR)—the number of choosing the same option across all repetitions (Alós-Ferrer & Garagnani, 2021; Sen, 1993). The desire to behave consistently is a powerful determinant of human behavior in repeated situations. Consistent behavior allows one to signal one’s personal and intellectual strength (Falk & Zimmermann, 2011), thereby resulting in a strong motivation to maintain self-image. From the computational perspective, results from a self-consistent Bayesian model in a perceptual decision task suggest that one’s perception is influenced by both the present sensory evidence and previous decision history (Luu & Stocker, 2018). When it comes to morality, maintaining consistency is crucial to ensure that our decisions align with our values, beliefs, or self-image (Jefferson, 2020; Xu et al., 2024). In a previous study, participants were typically presented with the same or similar situations repeatedly (Garrett et al., 2016), but the effect of self-consistency has not been investigated.

Reward, self-referential thinking and cognitive control are closely linked to moral decisions, especially in the case of honest/dishonest behavior (Speer et al., 2022a). Functional magnetic resonance imaging (fMRI) studies have shown that social norm-based intrinsic rewards are encoded in the nucleus accumbens (NAcc) and caudate—the brain regions responding to external rewards such as monetary gains, thereby providing direct evidence for the existence of an intrinsic reward system for social norms (Abe & Greene, 2014; Knutson et al., 2001; O’Doherty et al., 2002). However, the pursuit of self-interest can lead to moral norm conflict because people often perceive self-serving behavior as morally wrong (Peterson, 1996). Thus, people sometimes perform dishonest acts consciously and deliberately by trading off between the expected external benefit and the moral cost of the dishonest acts (Allingham & Sandmo, 1972; Becker, 1968). When considering whether to lie or not, cognitive control is needed to override one’s moral default (Speer et al., 2020). Specifically, deceivers exhibit increased activation in the cognitive control brain regions, such as the anterior cingulate cortex (ACC) and inferior frontal gyrus (IFG), and greater activity in the nucleus accumbens (NAcc) when making honest decisions. On the other hand, people who are generally honest exhibited higher activity in the self-referential thinking network (posterior cingulate cortex (PCC), the bilateral temporo-parietal junctions (TPJs), and the ventromedial prefrontal cortex (vmPFC)) to match the actions in the present moment with their values (Speer et al., 2022a).

In complex moral choices where external reward conflicts with the internal social norm, no option is dominant. Mouse tracking (MT) has been implemented as a powerful tool to measure real-time mouse movements (Freeman et al., 2011), providing information regarding the time course of the dynamic competition process. It has been applied to the detection process of dishonesty or untruthful responses (Duran et al., 2010; Monaro et al., 2017). In previous studies, researchers mainly applied descriptive and quantitative analyses, such as examining the overall shape, direction changes, and deviations from a straight path, or calculating the movement duration, path length, curvature, velocity, and smoothness of the trajectories (Freeman & Ambady, 2010; Kieslich & Hilbig, 2014; Spivey et al., 2005). Among these, the area under the curve (AUC)—the geometric area between the actual trajectory and the ideal straight path—is one of the most widely used indices in binary decision-making paradigms (Freeman & Ambady, 2010; Freeman et al., 2013; Hehman et al., 2014; Lazerus et al., 2016; Maldonado et al., 2019). We selected AUC as our primary index because this measure is theoretically grounded in dynamic decision frameworks showing that AUC is a proxy of response conflict strength and reflects the difference of subjective value between two options (Stillman et al., 2018, 2020). Higher AUC values may indicate higher conflict between choices, whereas lower AUC values may suggest more straightforward decision-making processes (Stillman et al., 2020). Thus AUC offers a single continuous summary of motor uncertainty that correlates strongly with both reaction time and neural conflict signals (Hehman et al., 2014).

Throughout the time course of a decision, our weighting of options is rapidly evolving and changing (Krajbich et al., 2010). This dynamic process results in the experience of difficulty in making a choice. Thus, we define conflict as the lack of consistency of the tendency toward one option. In formulating this definition of conflict, we draw on the drift–diffusion model (DDM), which has been implemented to decompose different attributes in value-based decision making (Busemeyer & Johnson, 2004; Krajbich & Rangel, 2011; Shinn et al., 2020; N. J. Sullivan & Huettel, 2021). The DDM assumes that individuals accumulate evidence according to a relative value signal of two options until the evidence reaches a predetermined threshold. Within this framework, we are able to disassociate how individuals evaluate various factors in moral decisions.

So far, we still lack a comprehensive neurocomputational account of choice consistency in moral decisions. First, most studies focus on the consistency of the trial-by-trial response, such that researchers consider and find the effect of the switching cost of the preceding choice (Luu & Stocker, 2018; Weber et al., 2023), but not tracked in multiple runs. Secondly, the effect of the choice history of a certain item has rarely been quantified in an explicit way (Luu & Stocker, 2018), as the information of the response history has not been presented to the participants and utilized as a variable to consider in their decisions. Here, we used a fiber-optic computer mouse in the fMRI scanner, where participants were required to make repeated moral decisions. The correct answer, reward magnitude, and CR (how many times an option has been chosen) of each choice were explicitly marked to yield interpretable spontaneous moral decisions. We aim to investigate (1) how the human brain integrates information from past decisions and current rewards to make moral decisions, (2) how consistency and reward consideration are associated with dishonesty-related brain activities.

## 2 Methods

The study was not preregistered. All analyses were theory-driven and based on prior work in value-based decision making (Maier et al., 2020; N. Sullivan et al., 2015) and dishonesty research (Garrett et al., 2016; Speer et al., 2020, 2021, 2022a, 2022b). The complete analysis pipeline and code are openly documented to promote transparency.

### 2.1 Participants

All experimental procedures were approved by the institutional review board of the University of Macao (BSERE21-APP005-ICI). Regarding human participant recruitment, participant eligibility was first examined using an online pre-screening survey and determined by the experimenter using another detailed participant screening form. Exclusion criteria included claustrophobia, probability of pregnancy, history of heart disease and brain trauma, and the existence of any metal implant in the body.

The sample size for the fMRI study was comparable with the previous fMRI studies on dishonesty (e.g., n = 28 (Garrett et al., 2016), n = 40 (Speer et al., 2020), n = 25 (Sun et al., 2015), n = 31 (Pornpattananangkul et al., 2018)). We recruited 34 participants (16 females, mean age 20.44 years, SD = 2.29) via online advertisement from the University of Macau for the task. The local ethical review committee approved the experimental protocol. Participants were right-handed with normal or corrected-to-normal vision and had not participated in similar studies. All participants were right handed as confirmed through verbal inquiry and observation during task preparation. Each participant signed an informed consent form before the formal experiment, and the experimental protocol was approved by the local UM ethical review committee. At the end of the study, the participants were paid 130–150 MOP.

### 2.2 Experimental procedure

Stimuli were presented by Psychopy 2.3 (Peirce, 2007) standalone. Participants viewed a 17-inch MR-compatible LCD monitor (resolution: 2560 × 1600; refresh rate: 60 Hz) positioned at the end of the bore, approximately 40 cm from their eyes. The display was visible via a helmet-mounted, front-silvered mirror. An MRI-compatible optical mouse was used for participants to do the task in the scanner. Meanwhile, mouse trajectory was recorded during all task sessions using PsychoPy 2.3 (Peirce, 2007), with a sampling rate of 60 Hz.

**Fig. 1.**
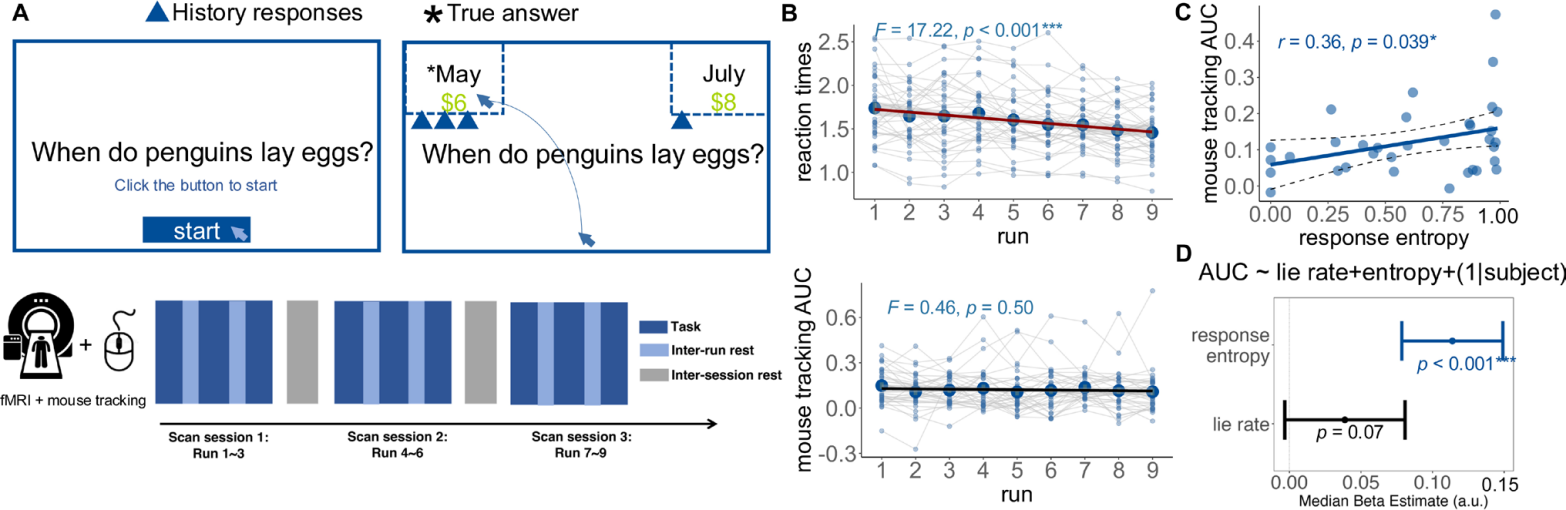
Illustration of the experimental paradigm and behavioral results. (A) The task procedure. The participants were presented with trivial questions, followed by two answers in the top left/right corner, respectively, with different amounts of reward. They were asked to deliver one answer to another person to gain more rewards. For the example trial, the left answer (with *) was correct but assigned a lower reward coin (“6”), whereas the other answer was incorrect, but assigned a higher reward (“8”). The entire task comprised three scanning sessions, each including three runs. (B) Reaction times decreased linearly through the runs ($F_{\left(1,304\right)}$ = 17.22, p<0.001 , $\eta_{p}^{2}$ = 0.054; upper panel), but the MT metric, area under the curve (AUC), did not change ($F_{\left(1,304\right)}$ = 0.46, p = 0.50, $\eta_{p}^{2}$ = 0.0015; lower panel). (C) AUC was positively correlated with response entropy (r=0.36 , p=0.039 , 95% CI from 0.021 to 0.62). (D) AUC was only correlated with response entropy (response entropy: β = 0.11, s.e. = 0.02, p<0.001 ; lie rate: β = 0.04, s.e. = 0.07, p<0.001 )

The experiment was a self-paced information transmission task with an MRI-compatible mouse inside the MRI scanner. Participants were asked to deliver information to another person nine times who was playing a trivia game where players answer questions across various categories. As shown in Figure 1A, the task comprised 9 runs, and each run consisted of 20 trivial questions (e.g., When do penguins usually lay their eggs? detailed in Supplementary Table S1) presented in the middle of the screen. Therefore, each question was repeated nine times. For every trial, the question was presented in the middle of the screen, and the start button was located at the bottom at the beginning of every trial (e.g., When do penguins usually lay their eggs? see Fig. 1A left panel). Participants were free to press the start button when they were prepared. Once they pressed the start button, correct answer (May) and incorrect answer (July) were shown in the left and right corners of the screen (Fig. 1A right panel). In addition, each choice was accompanied by other information, including which was the correct answer (marked with a black asterisk), the monetary reward, and the times the option had been chosen for the same question item (marked as blue triangles and only shown in runs 2 −9) (Fig. 1A right panel). Participants were informed of the meaning of this information prior to the task (see Supplementary Materials for detailed instruction for participants). To induce participants to choose between reward and honesty, we set more money for the incorrect answer in over 50% of the trials. Participants had 4 s to answer by moving the computer mouse, and feedback was shown after their choice and lasted for 1 s. If participants failed to respond within 4 s, warnings were displayed, and that trial was excluded from further analysis. After participants finished each session, the cumulative monetary reward in the session was displayed on the screen, and they were able to take a rest until they were ready to continue. Crucially, once participants started the trial, the position of the mouse was automatically initiated at the middle and bottom of the screen.

This paradigm is based on well-established “(dis)honesty” tasks used in prior research (Caspar, 2021; Kim & Kim, 2021; Yin et al., 2017); the present paradigm was designed to avoid the influence of decision consequence or interaction with mind inference. We did not provide information sending results and the partner’s choice as feedback.

Before the formal task in the scanner, participants went through a training phase, which aimed to familiarize them with the experimental procedure. To avoid a situation in which participants only sought money and ignored the importance of being honest, they were told that it was a knowledge transmission task wherein the option they chose would be the preference for the latter participants to win the money. We told participants that the followers would trust them and would not know their choices were involved in conflicts of interests, motivating them to keep consistent (see detailed instruction in Supplementary Materials). To ensure incentive conflict, we set a greater reward for the error answer in over 50% of the trials. At the end of the experiment, the payments were calculated as the cumulative reward for the task. This procedure encouraged participants to treat each trial as if it may be one that would count toward their monetary payment. After the formal task, participants completed two questionnaires, which consisted of the Interpersonal Reactivity Index (IRI; Davis, 1980), Rosenberg Self-Esteem Scale, and social value orientation (SVO; Murphy et al., 2011). The IRI scale is widely used for measuring individual differences in trait empathy and consists of four domains: (1) perspective taking (PT), (2) fantasy (FS), (3) empathetic concern (EC), and (4) personal distress (PD). SVO is used to measure one’s individual preferences during social interactions (Pletzer et al., 2018; Qi et al., 2018, 2020). Self-esteem was included because prior research suggest that individuals with higher self-esteem tended to show stronger motivation to maintain moral self-concept (Aquino & Reed II, 2002; Jordan et al., 2015).

### 2.3 Power and sensitivity analysis

To evaluate the sensitivity of our design, we conducted analyses to determine the minimum detectable effect sizes that could be reliably identified with 80% power at α=.05 given the current sample size (n=34 ).

Following recommendations by Button et al. (2013), we report sensitivity rather than post hoc power, as the latter yields biased estimates of achieved power.

For correlation analysis, we used the R package *pwr* (Champely et al., 2017). The results indicated that the present design provides 80% power to detect correlations of |r|≥0.46 (two-tailed α=.05 ), corresponding to medium-sized effects by conventional benchmarks (Cohen, 2013).

For linear mixed-effects models (LMMs), we performed simulation-based sensitivity analysis using the R package *simr* (Green & MacLeod, 2016). Fixed-effect coefficients for each predictor were systematically varied within plausible ranges, and 100 Monte Carlo simulations were performed per value to estimate the proportion of significant results at α=.05 . In the first LMM in the behavioral analysis (AUC ∼ lie rate + entropy + (1 |sub_nr)), we systematically modified the effect size of *entropy* (β=0.05–0.20 , step = 0.05 ) and *lie rate* (β=0–0.06 , step = 0.02 ), and performed 100 Monte Carlo simulations per value to estimate the probability of detecting a significant effect at α=0.05 . The results showed that for *entropy*, an effect of β≈0.055 successfully achieved approximately 80% power under the current sample size and random-effects structure (Supplementary Fig. S3 left panel). For lie rate, although the observed coefficient did not reach significance, the sensitivity analysis indicated that effects smaller than β≈0.06 could not be reliably detected with 80% power at the current sample size (Supplementary Fig. S3 right panel), suggesting that the non-significant result may reflect limited design sensitivity. In the second LMM (isLie ∼ dishonest CR + relative reward + relative CR + (1 | subject)), we systematically modified the effect size of relative CR (β=0.01–0.05 , step = 0.005 ), *relative reward* (β=0.035–0.055 , step = 0.005 ), and *dishonest CR* (β=0.0025–0.01 , step = 0.0025 ), and performed 100 Monte Carlo simulations per value to estimate the probability of detecting a significant effect at α=0.05 . The results showed that for *relative CR* and *relative reward*, the effects within the tested range successfully achieved approximately 100% power under the current sample size and random-effects structure (Supplementary Fig. S4 left and middle panels). For dishonest CR, although the observed coefficient did not reach significance, the sensitivity analysis indicated that effects smaller than β≈0.0125 could not be reliably detected with 80% power at the current sample size (Supplementary Fig. S4 right panel), suggesting that the non-significant result may reflect limited design sensitivity.

For the mediation analysis, we used the R package semTools (Jorgensen et al., 2016) to simulate repeated samples under the fitted mediation model and obtain Monte Carlo confidence intervals for the indirect effect (a×b ). The estimated indirect effect was 0.96 , 95% Monte Carlo CI [0.19 , 1.90 ], which excludes zero, confirming that the mediation effect is robust at the current sample size (n=34 ). The corresponding total effect was 1.25 [0.21 , 2.37 ]. These results indicated that the present design has sufficient sensitivity to detect indirect effects of this magnitude.

### 2.4 Computation of response entropy

Entropy (Shannon, 1948) is one way to quantify the randomness of a system, which is adopted to quantify the choice consistency. After excluding the timeout trials, the entropy of choices is defined as the entropy of a Bernoulli process in which binary choices are generated with probability p and 1 – p (S. Wang et al., 2023). Specifically, the choice entropy can be written as a function H(p) of the probability that participants lie, p. A higher entropy indicates a lower response consistency.

$$\begin{array}{c} H(p)=-p{\text{log}}_{2}p-(1-p){\text{log}}_{2}(1-p). \end{array}$$ (1)

As shown in Supplementary Figure S1, entropy is lowest when participants always tell the truth or always lie (predictable responses) and highest when they lied in a half of the responses (inconsistent behavior).

### 2.5 Mediation analysis

We tested a mediation model in which AUC predicted response entropy indirectly through the weight of relative reward, using the lavaan package in R (Rosseel, 2012). Path a estimated the effect of AUC on the weight of relative reward, path b estimated the effect of the weight of relative reward on response entropy, and path c’ represented the direct effect of AUC on response entropy after accounting for the mediator.

### 2.6 Mouse trajectory preprocessing

We extracted mouse trajectories from each session for each participant. Standard mouse-tracking preprocessing was conducted temporally and spatially (Freeman & Ambady, 2010; Xu et al., 2025). In a typical binary choice design, trajectories end at either the left or the right response option. For those analyses where the overall spatial direction is irrelevant, all trajectories were remapped so that they would end on the same side. We used the R package “mousetrap” (Kieslich & Henninger, 2017) to map the trajectories to the left by default, suggesting that trajectories that end on the right-hand side are flipped from right to left. We rescaled all mouse trajectories into a standard coordinate space (top left: [-1, 1]; top right: [1, 1]) so that the cursor always started at [0, 0] (N. Sullivan et al., 2015; Xu et al., 2025). Temporally, time normalization was applied to the trajectories such that the duration of each trial was divided into 101 identical time bins using linear interpolation to obtain the average of their length across multiple trials (Dale et al., 2007; Duran et al., 2010; Freeman et al., 2008, 2010; Spivey et al., 2005; N. Sullivan et al., 2015).

### 2.7 Hierarchical drift–diffusion modeling

We used a Bayesian hierarchical drift–diffusion model (HDDM) to examine trial-by-trial parametric modulations of reward and cumulative history responses (CR) on latent decision processes (Wiecki et al., 2013). The DDM assumes that individuals accumulate evidence according to a relative value signal of two options until the evidence reaches a predetermined threshold. DDM was characterized by three key parameters: the drift rate (v), reflecting the average speed and direction of evidence accumulation; the threshold (a), representing to what extent the evidence accumulation will stop; and the starting point (z), indicating initial bias toward one option.

We examined the extent to which evidence accumulation in the decision making of IST was driven by relative reward and relative CR between choice options. To this end, we modeled choices and RT with a multi-attribute DDM in which choice resulted from the noisy accumulation of a relative value signal that applied linear weights to reward and CR. Rewards and CR were both normalized. We compared seven models (see Supplementary Fig. S8). In all of these models, the threshold was modulated by session number. This was to account for the significant reduction in RTs along the runs in IST (Fig. 1B). Moreover, in all of these models, the drift rate was weighted by relative reward and relative CR in a trial-by-trial manner. From Model 1 to Model 4, the session number modulated the weight of the drift rate of neither (M1), the relative CR (M2), relative reward (M3), or both relative CR and relative reward (M4). In Model 5, we added the session-dependent initial bias term (z) to Model 4. In Model 6, we tested whether participants were affected by the choice of the last run before the decision-making process, so we added the history response into the initial bias. In Model 7, we added the history response to the drift rate to test whether it could improve the fitting. Finally, Model 5 outperformed all the models according to the deviance information criterion (DIC; see DDM structure and DIC in Supplementary Fig. S8). To evaluate the goodness-of-fit of the favored model (Model 5), we simulated choices and RT based on the model parameters estimated from Model 5. Both the observed choice proportion and RT were within the 95% confidence interval of the model predicted values, for all sessions (Supplementary Table S2).

A Bayesian estimation procedure was adopted to estimate the joint posterior distribution of model parameters based on observed decision data (i.e., reaction times and choices). This framework assumes that individual participants are random samples drawn from group-level distributions. Following a standard procedure of HDDM model estimation, we used Markov chain Monte Carlo sampling methods for Bayesian approximation of the posterior distribution of parameters (generating 20,000 samples, discarding 1000 samples as burn-in Rollwage et al. (2020), with a thinning factor of 5). Here, dishonesty responses were coded as 1 and honesty choices were coded as 0. RTs longer than 4 s or shorter than 0.3 s were excluded (Yu et al., 2021). We inspected traces of model parameters, and their autocorrelation, and computed the R-hat (Gelman-Rubin) convergence statistics to ensure that the models had properly converged (Wiecki et al., 2013; Supplementary Table S3). Five chains were run, each with 11,000 iterations and 1000 burn-in samples. No R-hat statistics were larger than 1.1, indicating good convergence (Ulrichsen et al., 2020). Parameter distributions at group level and individual level are simultaneously estimated. Deviance information criterion (DIC), suitable for hierarchical model comparison, was used as a measure of goodness-of-fit (Wiecki et al., 2013). To evaluate whether the preferred model can reproduce key patterns in the observation, we carried out posterior predictive checks, where we simulated data based on the parameters derived from the preferred model. This analysis showed that the preferred model satisfactorily reproduced the observed proportion of dishonesty choices and the means, and the quantiles of RT (Supplementary Table S2). This indicates that the preferred model could reliably reconstruct the patterns in the observed data.

### 2.8 fMRI general linear model (GLM) analysis

Preprocessing of fMRI data was performed using fMRIPrep version 20.2.1 (Esteban et al., 2019) (see Supplementary Materials). The output images were used in the following analysis.

A GLM was constructed for each run and each participant. The time at which the stimuli showed up was treated as the onset time. The MT index, the AUC of mouse trajectory of each trial was added as the parametric modulator to account for the height of the BOLD signal. The resulting GLM was convolved with SPM’s canonical hemodynamic response function. The model was corrected for temporal autocorrelations using a first-order autoregressive model, and a standard high-pass filter (cutoff at 128 s) was used to exclude low-frequency drifts. The resulting beta maps were smoothed using a 4-mm full width at half maximum (FWHM) Gaussian kernel. After that, they were masked using the anatomical mask generated by fMRIPrep for each session per participant. The beta maps of the parametric modulator estimated from the GLM were used in inter-subject representational similarity analysis (ISRSA described below).

### 2.9 ROI selection and beta extraction

We focus on nine ROIs spanning cognitive control network, self-referential thinking network, and reward network mentioned in Speer et al. (2022a) (see Fig. 6A). Specifically, the cognition control network comprises the dorsolateral prefrontal cortex (DLPFC), inferior frontal gyrus (IFG), and anterior cingulate cortex (ACC). Self-referential thinking network comprised posterior cingulate cortex (PCC), and bilateral temporo-parietal junctions (TPJs). The reward network consisted of nucleus accumbens (NAcc) and caudate nucleus.

Specifically, the masks of DLPFC, PCC, and IFG were derived from Brodmann atlas, while the masks of precuneus, caudate, and ACC were derived from AAL3 atlas (Rolls et al., 2020). For NAcc, we used the Individual Brain Atlases using Statistical Parametric Mapping Software (IBASPM) within the WFU PickAtlas toolbox (Maldjian et al., 2003). Right TPJ mask was taken from Bzdok et al. (2013) (https://neurovault.org/images/783440/), and left TPJ mask was taken from Lobo et al. (2023) (https://neurovault.org/images/783441/) onNeuroVault.

### 2.10 Neural pattern similarity

Neural activation pattern of eight keywords related to our experiment (reward, reward anticipation, cognitive control, moral, errors, moral, consistency, maintenance) was first obtained from the NeuroSynth platform as a canonical reference. We then refined the mask using a criterion of a height threshold of P<0.001 (z>3.0 ) (see Supplementary Fig. S2). Multi-voxel AUC activity patterns of each participant across nine runs were then separately extracted from the same mask defined above. And then we computed Pearson’s correlation coefficients for the respective multi-voxel patterns with the canonical reference map (Zhuang et al., 2022).

### 2.11 Inter-subject representational similarity analysis (IS-RSA)

We used intersubject representational similarity analysis (IS-RSA) (Nguyen et al., 2019; van Baar et al., 2019) in consideration of DDM parameters to identify regions of the brain that were responsible for reward and CR. The IS-RSA was performed in Python 3.9 using the NLTools package version 0.4.7 (http://github.com/ljchang/nltools).

We first obtained each participant’s activity map in the univariate analysis by averaging the GLM beta maps of the parametric modulator (the effect of AUC) across nine runs. We then extracted these subject-level beta maps of each ROI. Next, the inter-subject pairwise Pearson correlation was calculated by Scipy ’s Spatial module to carry out a similarity matrix for each ROI. For DDM’s parameter space, we also created an inter-subject dissimilarity matrix using the Euclidean distance between the drift rates of relative reward and relative CR across participants. Figure 5A visualizes the fitted weight of relative reward (v_reward) and weight of relative CR (v_CR) in a two-dimensional space. Each dot represented a participant with x-axis representing the value of v_CR and y-axis representing the value of v_reward.

Last, we computed Spearman’s rank-order correlations between each condensed ROI dissimilarity matrix and the condensed model dissimilarity matrix. This explores brain regions encoding the reward and CR evaluation. To obtain significance levels of the resulting Spearman’s rhos , we performed the permutation tests by shuffling the order of the observations in the model dissimilarity matrix 10,000 times and calculated the proportion of the permuted rhos that exceeded the true rho .

### 2.12 Functional connectivity analysis

To explore how the value signal of the ROIs interact during the decision-making process, we implemented an FC analysis using the CONN toolbox (Whitfield-Gabrieli & Nieto-Castanon, 2012) (version 21.a) (https://www.nitrc.org/projects/conn) for the beta maps of the regressor. We employed a standard pipeline by using the pre-processed fMRI data. In the denoising step, we used linear regression to remove the influence of the following confounding effects on the fMRI time course: (1) BOLD signal from the white matter and CSF voxels (5 components each, derived using the anatomical component-based correction (aCompCor) implemented using the ART toolbox), (2) 18 confounds by fMRIprep: 6 residual head motion parameters, global signal (the average signal within the brain mask), framewise displacement (the quantification of the estimated bulk-head motion calculated using formula proposed by Power et al. (2012)), 6 additional noise components calculated using anatomical CompCor and 4 DCT-basis regressors, (3) effect of task condition using separate run regressors (for dishonesty and honesty conditions) convolved with the hemodynamic response function. Thus, we performed the connectivity analysis on the residuals of the BOLD time series after removing condition-related activation/deactivation effects (Fair et al., 2007; Finc et al., 2017; Whitfield-Gabrieli & Nieto-Castanon, 2012). Finally, the denoising step included temporal bandpass filtering (0.008–0.09 Hz), and linear detrending of the functional time course. Following pre-processing, we performed condition-dependent FC analysis on the mean BOLD time course (Whitfield-Gabrieli & Nieto-Castanon, 2012) extracted from the selected ROIs.

## 3 Results

### 3.1 Choice conflict is correlated with response entropy

We first examined behavioral indices to quantify consistency and its relationship with choice conflict. The participants were instructed to decide which answer to deliver while the other person had no information about the correct answer; thus, participants were motivated to choose the correct answer because their decision mattered for the other person. However, we assume that their decision could be biased by rewards. To induce dishonest responses, we set more rewards for error answers in more than 50% trials (Supplementary Fig. S5B). The dishonesty probability was computed as the ratio of selecting wrong answers. The behavioral results showed large individual differences in the levels of dishonesty (mean = 38.20%, median = 41.11%, SD = 18.7%; Supplementary Fig. S5A), so we mainly focused on the moral decision itself. Generally, lie rates were negatively correlated with social value orientation (SVO) (r=−0.44 , p=0.008 , 95% CI from -0.68 to -0.13; Supplementary Fig. S5C), suggesting that the manipulation of dishonest decision was successful.

RTs decreased linearly through the runs ($F_{\left(1,304\right)}$ = 17.22, p<0.001 , $\eta_{p}^{2}$ = 0.054; Fig. 1B upper panel), but the MT metric, area under the curve (AUC), did not change ($F_{\left(1,304\right)}$ = 0.46, p = 0.50, $\eta_{p}^{2}$ = 0.0015; Fig. 1B lower panel). The AUC we measured can reflect the combination of different conflicts, mainly reflecting the level of hesitation or disputes between the two choices. Larger AUC values are linked to greater conflict in response and more hesitation, and are usually accompanied by longer RTs. The stability of MT metric was also confirmed by the cursor velocity along the x direction (Supplementary Fig. S6). To depict the decision consistency, we adopted entropy (Abramson, 1963; Shannon, 1948; S. Wang et al., 2023) (see Section 2 for details) to quantify the randomness or inconsistency. Entropy is a natural measure of uncertainty in probability distributions (Shannon, 1948). Higher entropy characterized inconsistent choices for both honest and dishonest choices. We found that AUC was positively correlated with response entropy (r=0.36 , p=0.039 , 95% CI from 0.021 to 0.62; Fig. 1C), suggesting less consistent participants exhibited greater decisional hesitation. To examine whether stable AUC was related to more or less immoral behavior, we conducted a linear mixed model to predict AUC with lie rate and response entropy (AUC <− lie rate + entropy + (1 |subject)) and found that only response entropy could predict AUC (response entropy: β = 0.11, s.e = 0.02, p<0.001 ; lie rate: β = 0.04, s.e = 0.018, p<0.07 ; Fig. 1D).

### 3.2 The weight of relative reward mediates the relationship between choice conflict and response entropy

**Fig. 2.**
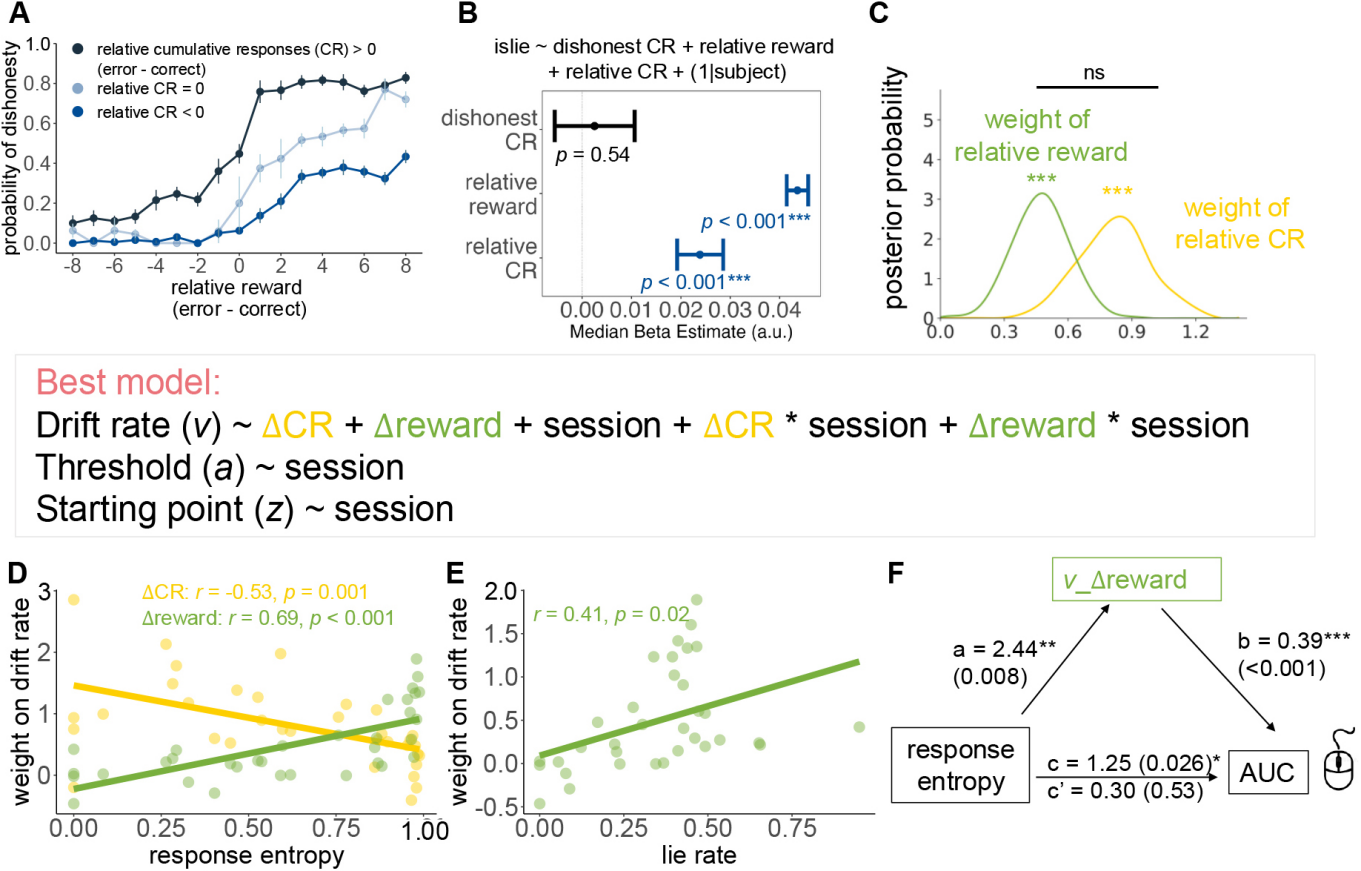
(A) The psychometric curve along the relative reward (error choice minus correct choice) under three conditions on the group level: more history dishonest responses (relative CR>0 ), more history honesty responses (relative CR<0 ), and same number of two responses (relative CR=0 ). We found that the history of choices shifted both the threshold and the total lie rate of the psychometric curve, indicating that more reward and more cumulative history responses of error choice led to a higher lie rate. (B) The result of a linear mixed model to test whether history dishonesty responses affect decision making. We found that only relative values of two choices contributed to dishonest responses (dishonest CR: β = -0.003, p = 0.54, 95% CI from -0.01 to 0.006; relative CR: β = -0.024, p<0.001 , 95% CI from -0.03 to -0.02; relative reward: β = -0.044, p<0.001 , 95% CI from -0.046 to 0.041). (C) Based on the preferred models, trial-by-trial decision variables (relative reward, relative CR) had significant impacts on the drift rate (all posterior probabilities = 1), and the weight of relative CR was larger than relative reward (posterior probability = 0.9965). (D) The weight of relative CR was negatively correlated with the overall response entropy (r = -0.53, p=0.001 , 95% CI from -0.74 to -0.23). The weight of relative reward was positively correlated with the overall response entropy (r = 0.69, p<0.001 , 95% CI from 0.46 to 0.83). (E) The weight of relative reward was positively correlated with the lie rate (r = 0.41, p = 0.02, 95% CI from 0.085 to 0.66). (F) There was a significant indirect effect of the weight of relative reward indicating that the positive relationship between the choice conflict and response entropy may partly be accounted for the weight of reward (a = 2.44, p = 0.008; 95% CI from 0.65 to 4.24; b = 0.39, p<0.001 ; 95% CI from 0.23 to 0.55; $c^{\prime }$ = 0.30, p = 0.53; 95% CI from -0.64 to 1.23; c = 1.25, p = 0.026, 95% CI from 0.15 to 2.36).

Having established that AUC was captured by entropy, we next used a hierarchical drift–diffusion model (HDDM) to uncover the latent cognitive processes underlying these effects. Apart from entropy quantifying consistency on subject level (S. Wang et al., 2023), we also quantify consistency on trial level with CR (Alós-Ferrer & Garagnani, 2021; Sen, 1993). To examine the effect of the reward and CR marked on the screen, we fitted the psychometric curve along the relative reward (error choice minus correct choice) under three conditions at the group level: more history dishonest responses (relative CR > 0), more history honesty responses (relative CR<0 ), and same number of two responses (relative CR = 0). We found that the history of choices shifted both the threshold and the overall lie rate of the psychometric curve, indicating that more reward and more cumulative history responses of error choice led to a higher lie rate (Fig. 2A). We also conducted a linear mixed model to test whether history dishonesty responses affect decision making (islie <− dishonest CR + relative reward + relative CR + (1 | subject)). We found that only relative values of two choices contributed to dishonest responses (dishonest CR: β = -0.003, p = 0.54, 95% CI from -0.01 to 0.006; relative CR: β = -0.024, p<0.001 , 95% CI from -0.03 to -0.02; relative reward: β = -0.044, p<0.001 , 95% CI from -0.046 to 0.041; Fig. 2B).

We next used a Bayesian hierarchical drift–diffusion model (HDDM) to examine trial-by-trial parametric modulations of reward and cumulative history responses (CR) on latent decision processes (Wiecki et al., 2013). We examined the extent to which evidence accumulation in the decision making of IST was driven by relative reward and relative CR between choice options. To this end, we modeled choices and RT with a multi-attribute DDM in which choice results from the noisy accumulation of a relative value signal that applies linear weights to reward and CR. Based on the winning model (M5), trial-by-trial decision variables (relative reward, and relative CR) had significant impacts on the drift rate (all posterior probabilities = 1), but the weight of relative CR was not significantly different from relative reward (posterior probability = 0.0575; Fig. 2C). Also, the correlation between the two parameters did not reach significance, but showed a negative trend (r = -0.34, p=0.052 , 95% CI from -0.61 to 0.002). Crucially, the weight of relative CR was negatively correlated with the overall response entropy (r = -0.53, p=0.001 , 95% CI from -0.74 to -0.23; Fig. 2D), validating the concept of CR and the structure of Model 5. Moreover, the weight of relative reward was positively correlated with the overall response entropy (r = 0.69, p<0.001 , 95% CI from 0.46 to 0.83; Fig. 2D) and the lie rate (r = 0.41, p = 0.02, 95% CI from 0.085 to 0.66; Fig. 2E). Finally, we tested a mediation model in which the weight of relative reward mediated the relationship between AUC (MT index of conflict) and response entropy. We found a significant total effect (c = 1.25, p = 0.026, 95% CI from 0.149 to 2.36), with a positive relationship between AUC and response entropy. More importantly, there was a significant indirect effect of the weight of relative reward indicating that the positive relationship between the choice conflict and response entropy may partly be accounted for by the weight of reward (a = 2.44, p = 0.008; 95% CI from 0.65 to 4.24; b = 0.39, p<0.001 ; 95% CI from 0.23 to 0.55; $c^{\prime }$ = 0.30, p = 0.53; 95% CI from -0.64 to 1.23; c = 1.25, p = 0.026, 95% CI from 0.15 to 2.36; Fig. 2F).

### 3.3 Precuneus tracked choice conflict

**Fig. 3.**
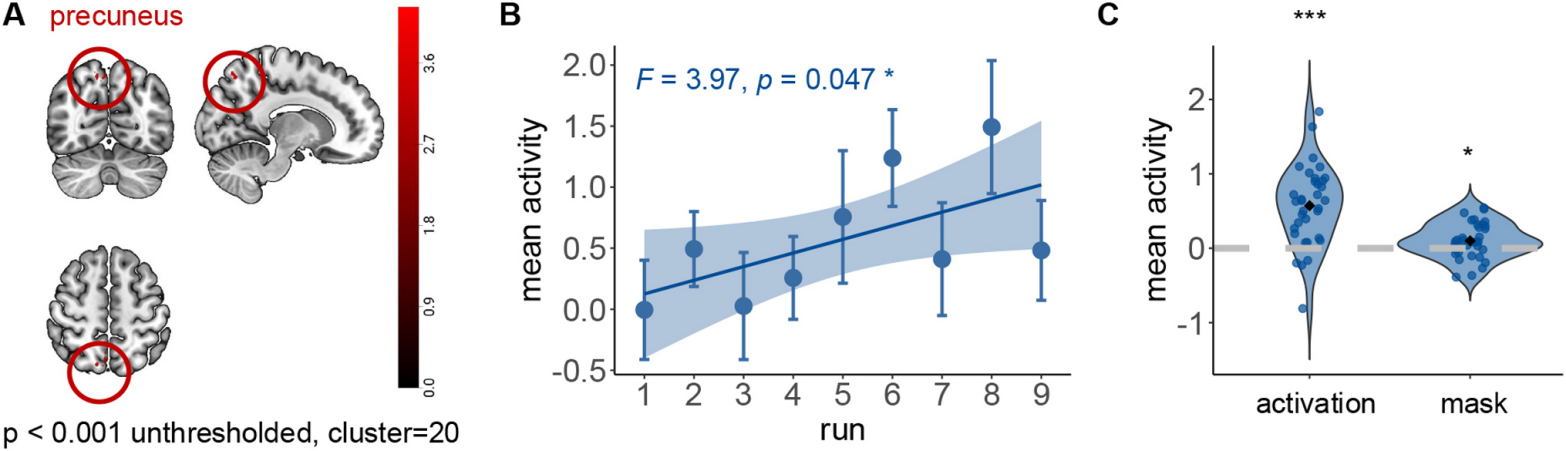
(A) The precuneus encoded AUC (x=12,y=−68,z=56 , p<0.001 unthresholded, cluster = 20); (B) and the activities increase across runs (F = 3.97, p = 0.047, $\eta_{p}^{2}$ = 0.013); (C) The activity of precuneus ROI was also significantly larger than 0 (t = 2.46, p=0.02 , 95% CI = 0.02 to 0.18). The “activation” denotes the regions identified from the modulation GLM analysis, and the “mask” denotes the precuneus region from the Brodmann atlas.

We next seek to identify the neural correlates of AUC. If a brain region represents regressors, then differential activity will be driven by processing effort and hence should positively correlate with RT (Taylor et al., 2014). We predict that greater decisional hesitation underlies stronger neural activity in related brain areas. Here we used AUC, which quantifies choice conflict, to account for the effort processing choice conflict. To track the neural effects of AUC (choice conflict), we performed an exploratory GLM where AUC served as parametric modulator (see Section 2). Averaging across all scan runs, we found activation in precuneus (x=12, y=−68, z=56 , p<0.001 cluster-level uncorrected, cluster = 20; Fig. 3A), and the activities increase across runs (F = 3.97, p = 0.047, $\eta_{p}^{2}$ = 0.013; Fig. 3B). The activation did not reach cluster-level FWE correction ($p_{FWE}$ = 0.092). Thus we also extracted the precuneus ROI from automated anatomical labeling (AAL) atlas (Rolls et al., 2020), and found that the activity was also significantly larger than 0 (t = 2.46, p=0.02 , 95% CI = 0.02 to 0.18).

### 3.4 AUC pattern change in reward ROIs predicted behavior patterns

**Fig. 4.**
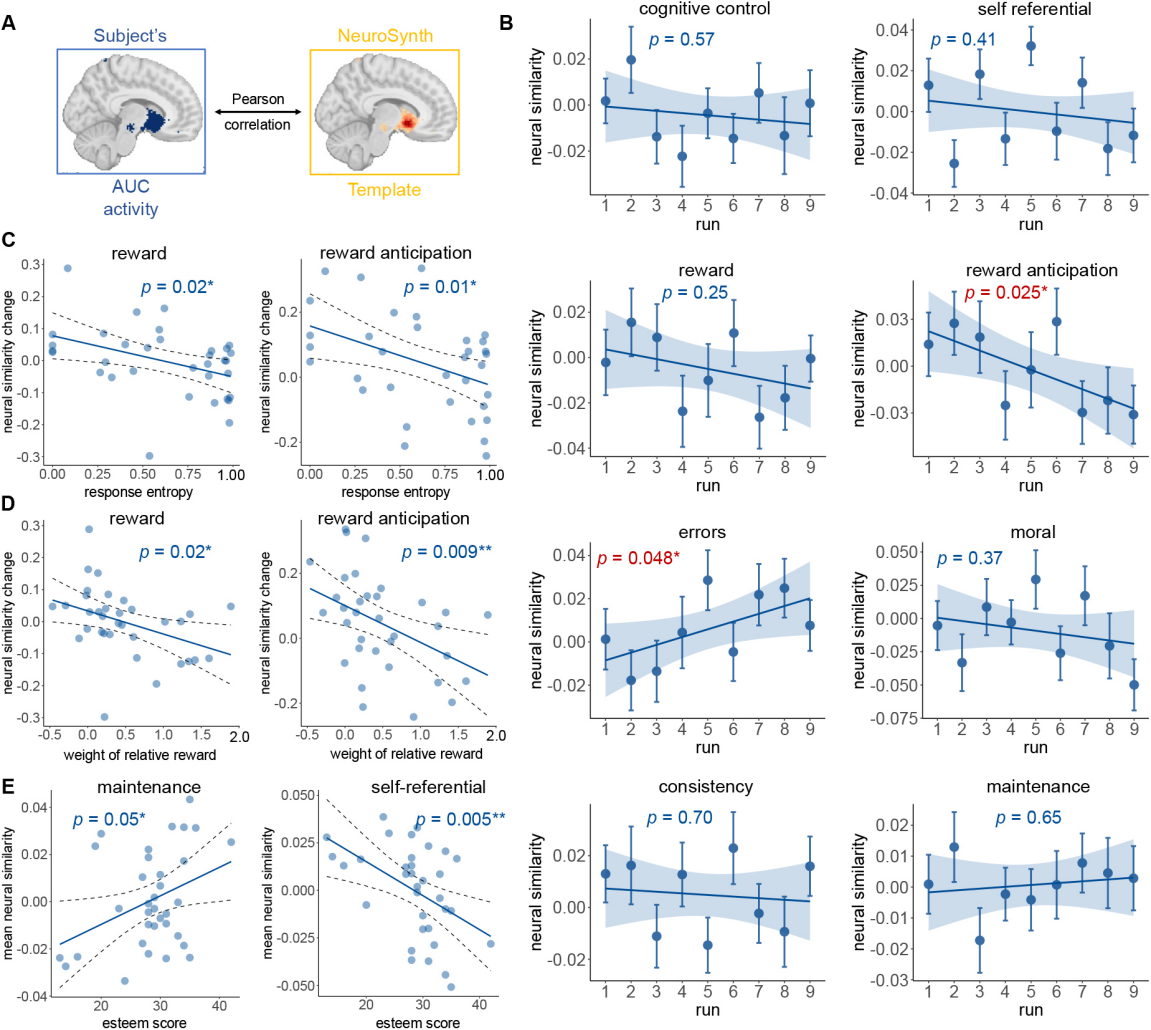
Dynamic changes of multivariate patterns of activity in NeuroSynth maps. (A) An illustration of multi-voxel pattern similarity analysis, by computing Pearson’s correlation of multi-voxel AUC modulation activity pattern (in blue) with the canonical dishonesty-related brain activation map derived from the NeuroSynth platform (in yellow). (B) The neural similarity to the “reward anticipation” pattern extracted from the NeuroSynth template decreased through runs ($F_{1,304}$ = 5.089, p = 0.025, $\eta_{p}^{2}$ = 0.016), while the neural similarity to “errors” increased ($F_{1,304}$ = 3.93, p = 0.048, $\eta_{p}^{2}$ = 0.013). (C) The decrease in neural similarity of reward and reward anticipation map from the first run to the last run was correlated with response entropy (reward: r = -0.41, p = 0.015, 95% CI from -0.66 to -0.088; reward anticipation: r = -0.42, p = 0.01, 95% CI from -0.67 to -0.10) and (D) the weight of relative reward (reward: r = -0.39, p = 0.02, 95% CI from -0.64 to -0.06; reward anticipation: r = -0.44, p = 0.009, 95% CI from -0.68 to -0.12). (E) Mean neural similarity to “maintenance” and “self-referential” predicted esteem score (maintenance: r = 0.37, p = 0.03, 95% CI from 0.035 to 0.63; self-referential: r = -0.47, p = 0.005, 95% CI from -0.70 to -0.15).

While univariate analysis identified precuneus encoding AUC, it did not capture whether the representation of AUC involved other information. Thus to investigate the multivariate patterns of AUC modulation effect, we selected several maps related to our research questions from NeuroSynth (see Section 2 and Fig. 4B). First, we conducted a similarity analysis for multi-voxel activity patterns of each run relative to the canonical reward anticipation-related activation map from the NeuroSynth. Such an approach allowed us to assess how the neural activity patterns of AUC across all runs were similar to the NeuroSynth meta-analysis of reward brain patterns. We found that the neural similarity to the “reward anticipation” pattern extracted from the NeuroSynth template decreased through runs ($F_{1,304}$ = 5.089, p = 0.025, $\eta_{p}^{2}$ = 0.016), while the neural similarity to “errors” increased ($F_{1,304}$ = 3.93, p = 0.048, $\eta_{p}^{2}$ = 0.013). These results indicate a greater involvement of errors and a less involvement of reward-related brain systems across runs.

Further, the decrease from the first run to the last run was correlated with response entropy (r = -0.42, p = 0.01, 95% CI from -0.67 to -0.10; Fig. 4C right panel) and the weight of relative reward (r = -0.44, p = 0.009, 95% CI from -0.68 to -0.12; Fig. 4D middle panel). We also found that mean neural similarity to “maintenance” and “self-referential” predicted esteem score (Fig. 4E; maintenance: r = 0.37, p = 0.03, 95% CI from 0.035 to 0.63; self-referential: r = -0.47, p = 0.005, 95% CI from -0.70 to -0.15).

To test whether the adaption of (AUC) choice conflict in reward ROIs was due to hand movement, we extracted mean activity from the hand movement mask on NeuroSynth (https://neurosynth.org/). We found that the activity of the hand movement mask did not change across the runs (F = 2.31, p = 0.13, $\eta_{p}^{2}$ = 0.008; Supplementary Fig. S7A). Also, the activity change from the first run to the last run did not correlate with the weight of relative reward (r = 0.075, p = 0.68, 95% CI from -0.27 to 0.40; Supplementary Fig. S7B), relative CR (r = -0.07, p = 0.71, 95% CI from -0.40 to 0.28), and response entropy (r = 0.002, p = 0.99, 95% CI from -0.34 to 0.34; Supplementary Fig. S7C).

### 3.5 Individual variation in IFG, ACC, and TPJ reflects differences in the weight of CR and reward

**Fig. 5.**
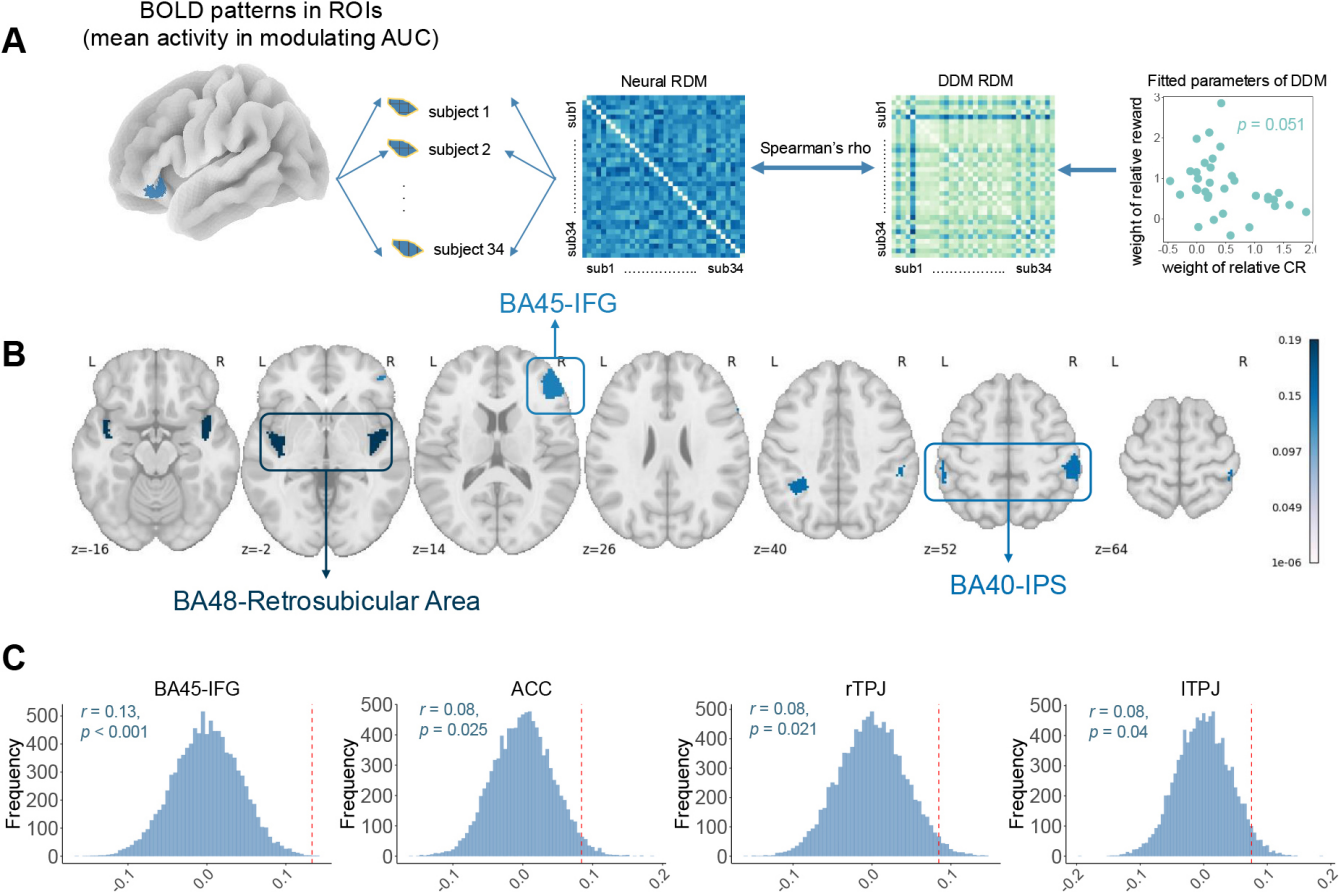
Patterns of choice conflict in brain represented consistency and reward together. (A) Illustration of inter-subject representational analysis (ISRSA). (B) ISRSA results of the whole brain. Significant inter-subject representational similarity effects in IFG, retrosubicular area, and IPS were observed. (C) ISRSA results of dishonesty-related ROIs. IFG, ACC, and bilateral TPJs were observed. The histogram represents the null distribution from permutation tests, and the red dashed line denotes the correlation coefficient (r).

For now, only limited AUC-related brain regions were identified in the univariate analysis reported above. One possibility is that the neural mechanisms underlying AUC may be encoded in a distributed manner. As AUC is an integrated index of relative reward and relative CR, we next examined how AUC modulation effect could reflect different preferences for consistency and reward. To this end, thus we conducted an inter-subject representational similarity analysis (ISRSA) to seek the brain regions encoding the evaluation of both the weight of relative CR and reward. We first created a geometric representational space of DDM parameter space (weights of relative CR and reward). We then searched for brain regions that showed a similar representational geometry to this distance measure in terms of the multi-voxel activity pattern correlations between each pair of participants (Fig. 5A). We used an a priori 200-parcel whole-brain parcellation based on meta-analytic functional coactivation of the NeuroSynth database (de la Vega et al., 2016), and we identified parcels that survived FDR correction (Fig. 5C). We observed significant inter-subject representational similarity effects in IFG, retrosubicular area, and IPS. We also conducted ISRSA separately on ROIs involved in Speer et al. (2022a), and also found that IFG, ACC, and bilateral TPJs encoded CR and reward together (Fig. 5C). These results indicated that AUC-related (conflict-related) activity patterns in cognitive control and self-referential regions were more similar than in other brain regions across participants who shared similar weights on reward and consistency when making repeated moral choices.

### 3.6 Interactions among cognitive control, reward, and self-referential regions predicted behavior results

**Fig. 6.**
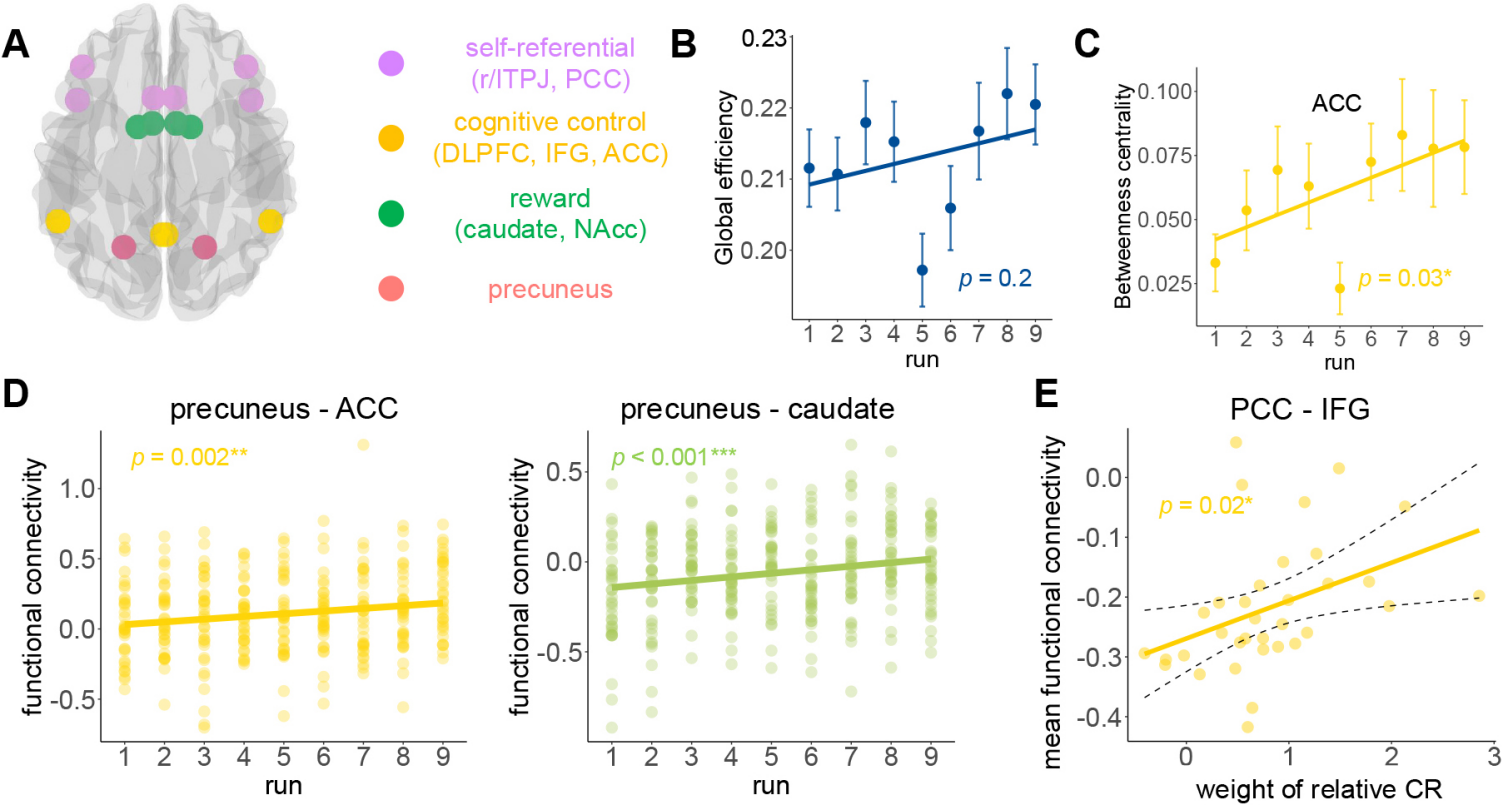
Results of the FC analysis. (A) Selected ROIs. (B) There was no significant linear change in global network efficiency ($F_{1,304}$ = 1.64, p = 0.20, $\eta_{p}^{2}$ = 0.005). (C) A general increase in betweenness for ACC was observed ($F_{1,304}$ = 4.87, p = 0.03, $\eta_{p}^{2}$ = 0.02), and a significant increase from run 1 to run 9 (t = -2.24, p=0.03 , 95% CI = -0.09 to -0.004). (D) The FC between precuneus and ACC/caudate increased along the runs (precuneus–ACC: $F_{1,304}$ = 9.31, p = 0.002, $\eta_{p}^{2}$ = 0.03; precuneus–caudate: $F_{1,304}$ = 12.42, p < 0.001, $\eta_{p}^{2}$ = 0.04). (E) The mean FC between PCC and IFG across nine runs was positively related to the weight of relative CR (r = 0.40, p = 0.02, 95% CI from 0.07 to 0.65).

Finally, to understand how these distributed regions interact as a coordinated moral-consistency network, we examined functional connectivity among the nine ROIs identified above. We tested whether there was task-related FC among the cognitive control, reward, and self-referential brain regions that could reflect the behavioral traits. Thus we select nine ROIs according to previous research (Speer et al., 2022a) and results (Fig. 6A) to examine the interactions among three sub-networks. Global efficiency was computed to assess how easily information flowed across a network via the shortest path between all pairs of nodes. Overall, we did not observe a significantly linear change in global network efficiency (Fig. 6B; $F_{1,304}$ = 1.64, p = 0.20, $\eta_{p}^{2}$ = 0.005). The betweenness centrality, which referred to the fraction of all shortest paths in the network that passed through a given node (Rubinov & Sporns, 2010), was computed to identify nodes that play a central role in coordinating with others over runs. These analyses revealed a general increase in betweenness for ACC (Fig. 6C; $F_{1,304}$ = 4.87, p = 0.03, $\eta_{p}^{2}$ = 0.02), and a significant increase from run 1 to run 9 (t = -2.24, p=0.03 , 95% CI = -0.09 to -0.004).

We then examined the connectivity between the precuneus and other ROIs, as the precuneus was the only region encoding AUC. We found that the FC between precuneus and ACC/caudate increased along the runs (Fig. 6D; precuneus–ACC: $F_{1,304}$ = 9.31, p = 0.002, $\eta_{p}^{2}$ = 0.03; precuneus–caudate: $F_{1,304}$ = 12.42, p < 0.001, $\eta_{p}^{2}$ = 0.04). Finally, we investigated FCs that could predict behavior results. We found that the mean FC between PCC and IFG across nine runs was positively related to the weight of relative CR (Fig. 6E; r = 0.40, p = 0.02, 95% CI from 0.07 to 0.65).

## 4 Discussion

**Fig. 7.**
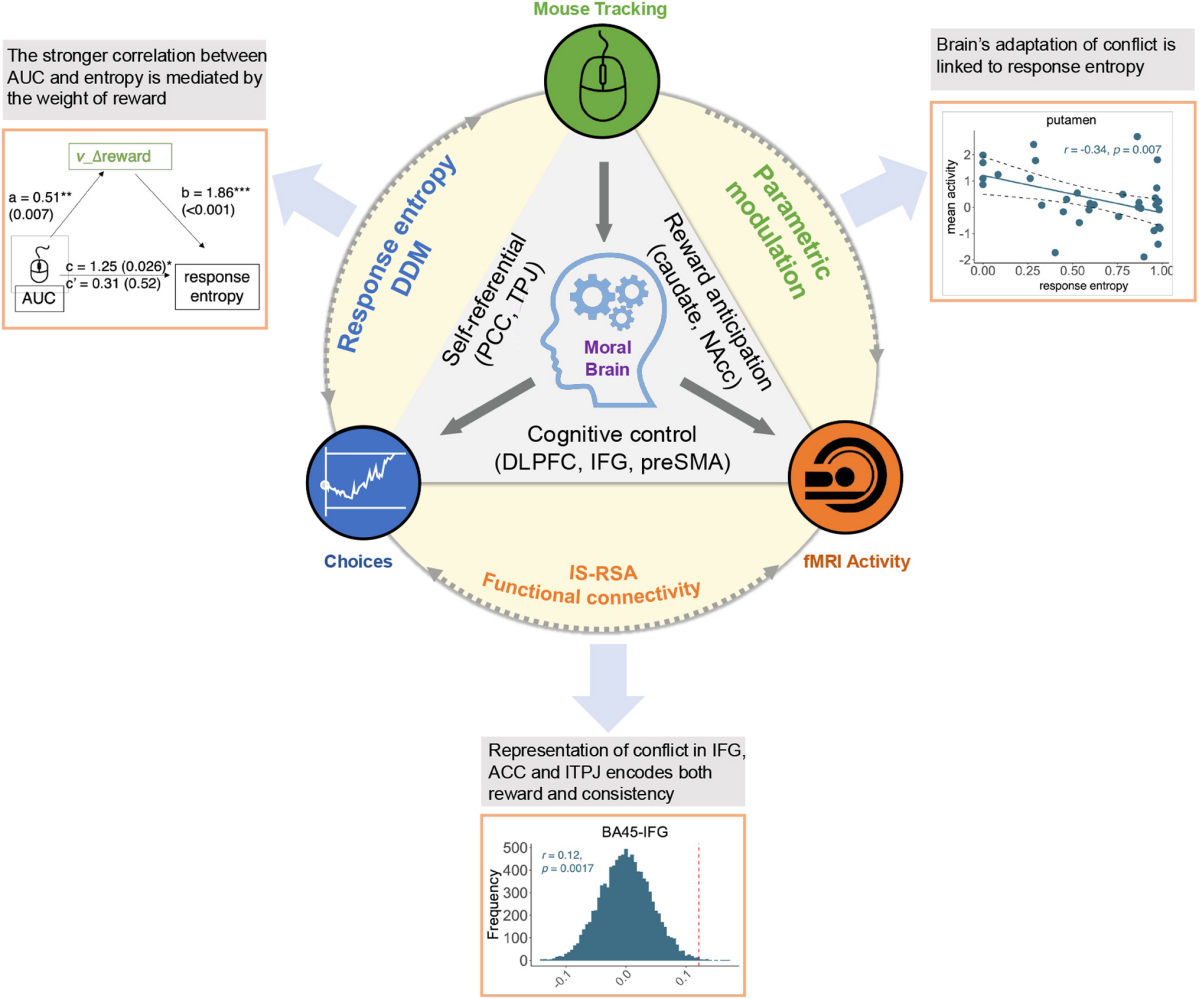
Our study utilizes an MRI-compatible mouse cursor to investigate how individuals make moral decisions with the consideration of CR and the current reward, with a simultaneous combination of MT and fMRI. We mainly adopted an ROI-based analysis, focusing on brain regions related to reward, cognition control, and self-referential. Through the neural representation of MT metric AUC, we explore the dynamics of choice conflict, which can predict choice entropy and the weight of consistency.

Our study utilizes an MRI-compatible mouse cursor (Fig. 1A) to investigate how individuals make moral decisions with the consideration of CR and the current reward, with a simultaneous combination of MT and fMRI (Fig. 7). Through the MT metric AUC and the activity in dishonesty-related ROIs (see Section 2), we explore the dynamics of choice conflict which is related to consistency at behavioral and neural levels. In combination with the weight of CR and reward obtained from DDM, we map the variation in reward and consistency onto the brain representation of choice conflict. Across individual- and group-level analysis, we find that AUC is related to response entropy rather than the lie rate. The modeling results show that individuals have a larger weight of CR over reward, which is correlated with response entropy. Also, the stronger link between AUC and response entropy was mediated by the weight of relative reward. The MT and fMRI results show that brain regions involved in dishonesty (cognitive-control, reward brain, and self-referential-related regions) adapt to choice conflict with decreased AUC modulation effect, and their representation of AUC reflects the evaluation of consistency and reward. Further, the brain connectivity between different ROIs also predicts response entropy and DDM parameters.

Overall, our findings show that brain regions associated with dishonesty adapted to the degree of choice conflict, showing reduced AUC modulation across runs. The distributed representation of AUC reflected the dynamic evaluation of both consistency and reward values. Furthermore, functional connectivity among these regions predicted both response entropy and the weight on reward and consistency, highlighting their coordinated role in integrating external incentives and internal consistency. The novelty of our research lies in combining explicit response history to (1) build a theoretical framework on moral decisions with considerations of both self-consistency and self-interest and (2) uncover how the evaluation between reward and consistency relates to the choice conflict, on the behavioral and neural level.

### 4.1 Evidence for the role of consistency during moral decisions

In the current binary decision task with an explicit presentation of the number of history responses and reward magnitude, we focused on the trade-off between maintaining consistency and seeking reward. We use response entropy and CR (how many times a choice has been chosen) to quantify consistency both on the subject level and trial level. Our results highlight the pivotal role of internal consistency in shaping moral decision dynamics (Figs. 1D and 2B, C). Beyond the pursuit of external rewards, individuals tended to align their current responses with prior choices (Fig. 2C), reflecting a drive for coherence across repeated moral judgments. Specifically, the weight of CR is negatively correlated with response entropy (Fig. 2D), indicating the validity of the modeling of CR. Further, the weight of relative reward is positively correlated with response entropy (Fig. 2D), suggesting the trade-off between consistency and reward. These results are plausible because the desire for self-interest can result in lying. For example, the study shows that self-serving dishonesty gradually increases with repetition (Garrett et al., 2016). A recent functional near-infrared spectroscopy (fNIRS) study reveals that the brain’s reward system remains active throughout the entire process of dishonest behavior, and show how the neural activity of reward system is related to subsequent dishonest behaviors (Liang et al., 2020). Our findings further suggest that such monetary reward-based manipulation is effective for motivated dishonesty (Mazar et al., 2008; Pang et al., 2022; Shalvi et al., 2011; Wu et al., 2021).

Although the reward plays an important role in moral decision making, participants show a larger weight of relative CR (Fig. 2C), such that a history of more dishonest responses makes them more likely to lie (Fig. 2A). This finding reflects that consistency is an important factor in modulating the lie rate. Decision history is an inescapable part of every similar or repeated decision, and heuristics offer individuals a general guide to follow (Scott et al., 2011; X. T. Wang, 2008). This is important because it suggests that groups are more likely to make dishonest decisions when there is a conflict between more history of dishonest responses and the more reward offered for the honest choice. Furthermore, over 80% of trials were designed to induce dishonesty, but a considerable number (about 50%) of participants only lied in approximately 50% of the trials (see Supplementary Fig. S5). These findings reveal an alternative explanation that despite seeking self-interest, consistency considerations play a pivotal role.

One limitation of the present paradigm is that it provided explicit reminders of prior responses and rewards, whereas in real-world moral contexts, such information is typically implicit. This intentional setting allowed us to isolate the computational and neural substrates of response history and reward information, but future work should examine whether similar dynamics emerge in more naturalistic moral settings.

### 4.2 Neural patterns of AUC predicted consistency and reward

Our findings reveal intriguing insights into the behavior and neural activity associated with AUC across various sessions. It is noted that although response time (RT) decreased linearly throughout successive scan runs, the MT metric AUC, which gauges the extent of choice conflict (Stillman et al., 2018, 2020), remained stable (Fig. 1B; Supplementary Fig. S6). Interestingly, the fMRI results revealed that precuneus stably encodes AUC. Previous studies revealed that precuneus exhibited increased activity while participants considered personal moral dilemmas (Greene et al., 2004; Huang et al., 2025). This suggests that the precuneus may play a consistent role in representing subjective conflict, even as participants become faster at making decisions. The different patterns between RT and AUC that RT decreases along the runs while AUC does not (Fig. 1B) imply that reduced motor hesitation does not necessarily reflect reduced cognitive conflict. RT primarily reflects the duration of decision execution (Ratcliff & McKoon, 2008; Wong & Wang, 2006), while AUC captures continuous competition between response alternatives (Freeman et al., 2011; Stillman et al., 2020). The sustained AUC representation in the precuneus across sessions further indicates that this region may support a stable internal evaluation of moral tension, regardless of habituation or learning effects. This aligns with recent accounts highlighting the precuneus as a key node in the default mode network involved in self-referential (Cavanna & Trimble, 2006). Together, these findings reinforce the notion that moral decision making engages distinct neural mechanisms beyond mere reaction speed, with the precuneus encoding conflict intensity in a temporally stable fashion.

Further, the neural pattern of AUC becomes less likely to reward anticipation map across runs (Fig. 4B). The decrease of neural similarity with the reward-related brain regions was correlated with factors such as response entropy and the weight placed on reward (Fig. 4C, D). This suggests that while early in the task AUC may partially reflect reward-driven value conflict, over time its representation becomes increasingly distinct from reward anticipation. One possible interpretation is that participants shift from reward-maximizing strategies to more rule-based or internally consistent modes of moral reasoning (Crockett, 2016; Hutcherson et al., 2015), leading to reduced engagement of classic reward-related NeuroSynth maps. The correlation with response entropy further implies that less change of reward-related activity in the last run was accompanied by more consistent decisions.

The ISRSA revealed that multivariate activity patterns in regions such as the IFG, ACC, TPJs, and retrosubicular cortex align with participants’ subjective trade-offs between CR and reward. These regions are known to support cognitive control and conflict monitoring in moral decision-making contexts (Caspar et al., 2020, 2021; Tricoche et al., 2024), as well as self-referential reasoning (Botvinick et al., 2004; Speer et al., 2022b), suggesting that people who emphasize either reward or consistency rely on partially distinct engagement of these networks when processing internal conflict. The involvement of bilateral TPJs, typically linked to social cognition and perspective taking (Carter & Huettel, 2013; Filmer et al., 2019; Qin & Wu, 2024; Saxe & Wexler, 2005; Schurz et al., 2014), points to a potential role of social-contextual reasoning in how consistency and reward are evaluated. Similarly, the consistent engagement of IFG across both whole-brain and ROI-based analysis supports the view that cognitive control mechanisms are tuned to individual moral preferences, adapting their processing architecture in accordance with stable decision parameters.

Together, these findings suggest that stable behavioral conflict signals can emerge from distinct and evolving neural processes. While initial conflict may partially rely on value-based computations, repeated moral decisions engage self-referential and control-related networks, with individual preferences shaping the representational architecture of conflict processing.

### 4.3 Moral decision tied to interaction among cognitive control, reward, and self-referential regions

The FC findings revealed how dynamic interactions among cognitive control, reward, and self-referential systems shaped moral decision making over time. To probe these interactions more directly, we examined FCs among nine a priori defined ROIs spanning cognitive control, reward, and self-referential systems (see Section 2). Our FC findings further underscored the central role of cognitive control in repeated moral decision making. Although global network efficiency did not change significantly across sessions (Fig. 6B), the increasing betweenness centrality of the ACC suggests that this region became increasingly central for coordinating information flow between subnetworks (Fig. 6B). Given the ACC’s established role in conflict monitoring and adaptive control (Botvinick et al., 2004), its rising centrality may reflect growing demands on integrating motivational and evaluative inputs as participants stabilize their decision strategies.

Crucially, precuneus—the only region found to stably encode AUC—showed increasing FC with both ACC and caudate nucleus, two nodes central to cognitive control and reward processing, respectively. This suggests a potential mechanism by which the precuneus integrates internally generated assessments of reward and consistency. Such cross-network integration may support the construction of consistent internal models for evaluating morally conflicting options, particularly as decisions become more self-guided over repeated exposure (Dixon et al., 2014; Spreng et al., 2010).

Moreover, FC between the PCC and IFG predicted the weight of relative CR, which aligned with the view that stable moral preferences are supported by functional links between self-referential and control networks (Denny et al., 2012; Hu et al., 2025; E. Wang et al., 2025; Zaki & Mitchell, 2011). Together, these results highlight that individual differences in moral decision making are not solely encoded in regional activations but are also reflected in the topology and evolving coordination of distributed neural systems.

Although the sample size of the current study (n=34 ) is relatively modest, it is comparable with that used in many recent fMRI studies investigating moral and value-based decision making (e.g., n = 28 (Garrett et al., 2016), n = 40 (Speer et al., 2020), n = 25 (Sun et al., 2015), n = 31 (Pornpattananangkul et al., 2018)). Our sensitivity analysis indicated that only correlations with |r| > 0.46 could reach 80% power given our sample size. While some of the observed correlations were below this threshold, their directions were consistent with our hypotheses, suggesting meaningful trends that warrant further examination in larger samples. To mitigate the risk of unstable estimates in the multivariate and connectivity analysis, several precautions were taken: (i) the p values of multivariate ISRSA (Fig. 5) were derived using permutation-based significance testing; (ii) connectivity analyses were restricted to a priori defined ROIs, thus minimizing the number of comparisons; and (iii) converging evidence of power analysis of correlation analysis in behavioral data has shown that the sample size is proper (see Section 2 for details). Nevertheless, we acknowledge that a larger sample would increase sensitivity to smaller effects and improve the generalizability of the results, which should be further examined in future research.

### 4.4 Combination of MT and fMRI helps reveal the decision dynamics

One novel aspect of this study is that we incorporated mouse tracking into the fMRI scanner, following previous studies (Hehman et al., 2014; Stolier & Freeman, 2017). Using the dynamic MT metric AUC reflecting conflicts and hesitation (Wu et al., 2021; Xu et al., 2025), we obtained evidence on increasing choice conflict associated with the trade-off between consistency and reward. This approach provided behavioral evidence of increased choice conflict associated with trade-offs between self-consistency and reward.

Although the absence of self-consistency does not necessarily indicate moral hypocrisy, our results indicate it is still a factor for moral decision (Monin & Merritt, 2012). Self-consistency contributes to moral decisions with a link to cognitive control brain regions such as IFG and ACC. The individual differences in moral trade-offs and fMRI signals further highlight the interaction between the cognitive control, self-referential, and reward brain, and potentially can inspire future studies on morality training processes. Extending this view, a deeper understanding of how humans balance external rewards and internal consistency during moral decision making may provide useful insights for the development of ethical frameworks in artificial intelligence (AI). Like humans, AI systems often encounter conflicts between maximizing external outcomes (e.g., performance, efficiency) and maintaining internally consistent ethical rules (Conitzer et al., 2017; Gabriel, 2020). A deeper understanding of decisions affected by external reward and internal consistency may help in the design of new types of AI ethic policy (S. Zhang et al., 2025). Indeed, governments and scientists may need effective intervention in the honesty of AI (Meinke et al., 2024) and a better understanding of how external and internal factors influence human moral behaviors.

## Supplementary Material

Supplementary Material

## Data Availability

Code for analysis can be found in our GitHub (https://github.com/andlab-um/RDdishonesty). Preprocessed fMRI data can be obtained from (https://doi.org/10.57760/sciencedb.CHNNeuro.00006), and the raw data are available from the corresponding author upon request.
