# Supplementary material for: Unraveling the neural basis of repeated moral decisions with mouse tracking and fMRI
Source: Imaging Neurosci (Camb). 2025 Dec 16;3:IMAG.a.1047. doi: 10.1162/IMAG.a.1047 (PMC12709548; doi:10.1162/IMAG.a.1047)
Supplement: Supplementary Material [file IMAG.a.1047_supp.pdf]

==

Supplementary materials

Detailed instruction for participants

Welcome to the game! This is a knowledge sending task, consisting of 20 obscure knowledge Q&A questions (multiple choice) over 9 rounds. You will provide reference answers for subsequent players, who are likely to refer to the information you provide. Subsequent players will only receive rewards if they choose the correct answer. After each choice, you will earn a certain number of coins, and the final number of coins determines your compensation. During the process, we will mark the correct answers (with the asterisk) and the coin count for two answers (in red). The blue triangle next to the score represents the answers you have chosen in previous rounds. If it differs from the answers chosen in previous rounds, it may confuse the next test-taker. We are now ready to start the first round.

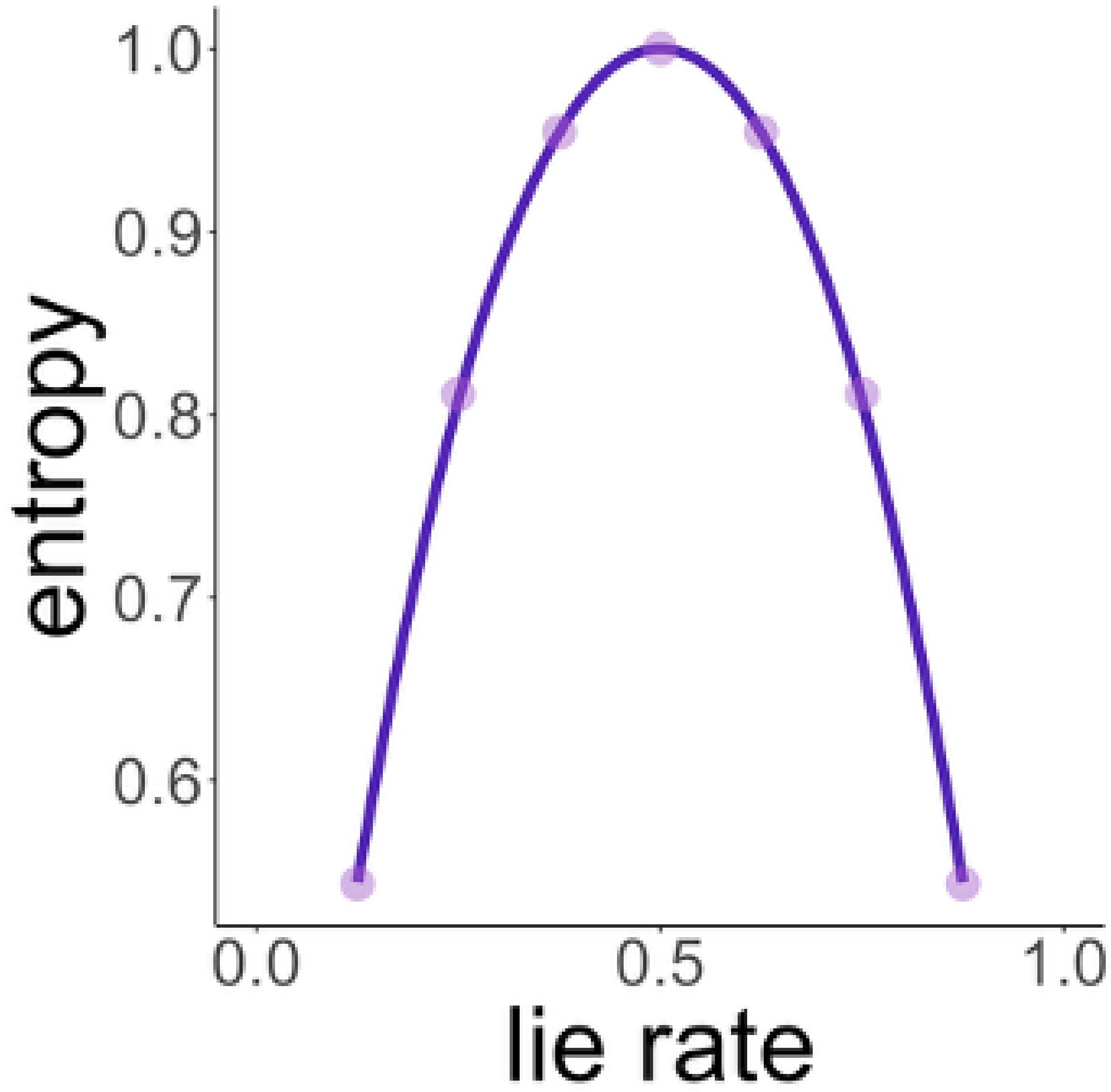

Fig. S1: **Entropy as a function of lie rate.**

fMRI acquisition

All fMRI data were acquired using a 3.0 T Siemens MAGNETOM Prisma MRI scanner with a 64-channel head coil in the Center for Cognitive and Brain Sciences, University of Macau. Neuroimaging data acquisition included the collection of both anatomical and functional data. High-resolution T1-weighted images were acquired for each participant (3D MPRAGE sequence; voxel size = 1 mm isotropic; FOV = 256 mm; 176 slices, slice thickness: 1.0 mm; TR = 2300 ms, TE = 2.26 ms, TI = 900 ms, flip angle = 8°). Whole-brain functional imaging was acquired by a T2*-weighted gradient echo, echo-planar pulse sequence in descending interleaved order with a 2.0mm slice gap, voxel size of 2.0 mm, slice thickness of 2.0 mm, TE of 30 ms, TR of 1000 ms and flip angle of 90°.

fMRI data preprocessing

The fMRI data preprocessing was based on Nipype (Gorgolewski et al., 2011), and was implemented in fMRIPrep v20.2.1 (Esteban et al., 2019) with the default pipeline.

Anatomical data preprocessing

A total of 1 T1-weighted (T1w) images were found within the input BIDS dataset. The T1-weighted (T1w) image was corrected for intensity non-uniformity (INU) with `N4BiasFieldCorrection` (Tustison et al., 2010), distributed with ANTs 2.3.3 (Avants et al., 2008, RRID:SCR_004757), and used as T1w-reference throughout the workflow. The T1w-reference was then skull-stripped with a *Nipype* implementation of the `antsBrainExtraction.sh` workflow (from ANTs), using OASIS30ANTs as target template. Brain tissue segmentation of cerebrospinal fluid (CSF), white-matter (WM) and gray-matter (GM) was performed on the brain-extracted T1w using `fast`(FSL 5.0.9, RRID:SCR_002823) (Zhang et al., 2001). Volume-based spatial normalization to one standard space (MNI152NLin2009cAsym) was performed through nonlinear registration with `antsRegistration` (ANTs 2.3.3), using brain-extracted ver-

sions of both T1w reference and the T1w template. The following template was selected for spatial normalization: *ICBM152 Nonlinear Asymmetrical template version 2009c*(RRID:SCR_008796; TemplateFlow ID: MNI152NLin2009cAsym) (Fonov et al., 2009).

Functional data preprocessing

For each of the 3 BOLD sessions found per subject (across all tasks and sessions), the following preprocessing was performed. First, a reference volume and its skull-stripped version were generated using a custom methodology of *fMRIPrep*. The BOLD reference was then co-registered to the T1w reference using `flirt`(FSL 5.0.9) (Jenkinson & Smith, 2001) with the boundary-based registration (Greve & Fischl, 2009) cost-function. Co-registration was configured with nine degrees of freedom to account for distortions remaining in the BOLD reference. Head-motion parameters with respect to the BOLD reference (transformation matrices, and six corresponding rotation and translation parameters) are estimated before any spatiotemporal filtering using `mcflirt` (FSL5.0.9) (Jenkinson et al., 2002). BOLD sessions were slice-time corrected using `3dTshift` from AFNI 20160207 (RRID:SCR_005927) (Cox & Hyde, 1997). The BOLD time-series were resampled onto their original, native space by applying the transforms to correct for head-motion. These resampled BOLD time-series will be referred to as *preprocessed BOLD in original space*, or just *preprocessed BOLD*. The BOLD time-series were resampled into standard space, generating a preprocessed BOLD session in *MNI152NLin2009cAsym space*. First, a reference volume and its skull-stripped version were generated using a custom methodology of *fMRIPrep*. Several confounding time-series were calculated based on the preprocessed BOLD: framewise displacement (FD), DVARS and three region-wise global signals. FD and DVARS are calculated for each functional session, both using their implementations in *Nipype* (following the definitions by (Power et al., 2014)). The three global signals are extracted within the CSF, the WM, and the whole-brain masks. Additionally, a set of physiological regressors were extracted to allow for component-based noise correction (*CompCor*) (Behzadi et al., 2007). Principal components are estimated after high-pass filtering the

preprocessed BOLD time-series (using a discrete cosine filter with 128s cut-off) for the two *CompCor* variants: temporal (tCompCor) and anatomical (aCompCor). tCompCor components are then calculated from the top 2% variable voxels within the brain mask. For aCompCor, three probabilistic masks (CSF, WM and combined CSF+WM) are generated in anatomical space. The implementation differs from that of Behzadi et al. in that instead of eroding the masks by 2 pixels on BOLD space, the aCompCor masks are subtracted a mask of pixels that likely contain a volume fraction of GM. This mask is obtained by thresholding the corresponding partial volume map at 0.05, and it ensures components are not extracted from voxels containing a minimal fraction of GM. Finally, these masks are resampled into BOLD space and binarized by thresholding at 0.99 (as in the original implementation). Components are also calculated separately within the WM and CSF masks. For each CompCor decomposition, the *k* components with the largest singular values are retained, such that the retained components' time series are sufficient to explain 50 percent of variance across the nuisance mask (CSF, WM, combined, or temporal). The remaining components are dropped from consideration. The head-motion estimates calculated in the correction step were also placed within the corresponding confounds file. The confound time series derived from head motion estimates and global signals were expanded with the inclusion of temporal derivatives and quadratic terms for each (Satterthwaite et al., 2013). Frames that exceeded a threshold of 0.5 mm FD or 1.5 standardised DVARS were annotated as motion outliers. All resamplings can be performed with *a single interpolation step* by composing all the pertinent transformations (i.e. head-motion transform matrices, susceptibility distortion correction when available, and co-registrations to anatomical and output spaces). Gridded (volumetric) resamplings were performed using `antsApplyTransforms` (ANTs), configured with Lanczos interpolation to minimize the smoothing effects of other kernels (Lanczos, 1964). Non-gridded (surface) resamplings were performed using `mri_vol2surf` (FreeSurfer).

Question sets

questions	correct answer	wrong answer
The representative works of Darwin's theory of evolution are	《On the Origin of Species》	《The Theory of the Gene》
The human genome contains	one billion genes	300 million base pairs
What is the unit of money in ancient times,"guan"?	thousand copper coins	Hundred copper coins
When do penguins usually lay their eggs?	May	July
What is Lu Xun's attitude towards Tagore?	disagree	agree
When did the Human Genome Project begin?	1985	1993
Which of the five senses responds the fastest?	taste	visual
What color is the top of the rainbow?	red	purple
Who was the first to propose democratic centralism?	Lennin	Satlin
"Groom" was first used to refer to	New Scholars	The new champion
What are tigers most afraid of?	tits poop	fire
The main components of the Martian atmosphere are	CO2	N2
Which of the following sovereign states does not have an army?	Andorra	Switzerland
What material are body armor made of?	Ceramic glass fiber reinforced plastic	Soft Impervious Steel
The concept of nanotechnology was introduced by	Richard	Verne
What elements are missing in people with "albinism"	Cu	Zn
Alpha Centauri is about 100 miles away from Earth	4.3 light years	30,000 light years

**questions**	**correct answer**	**wrong answer**
Saturn's moons have been detected at least	17	12
The size of the universe is approximately	15-20 billion light years	10 billion light years
The rotation period of Jupiter's equator is	9 hours 50 minutes	20 hours

Table S1: **Question sets for task.** These 20 uncommon questions were presented in every run randomly.

Neurosynth maps used in neural pattern similarity analysis

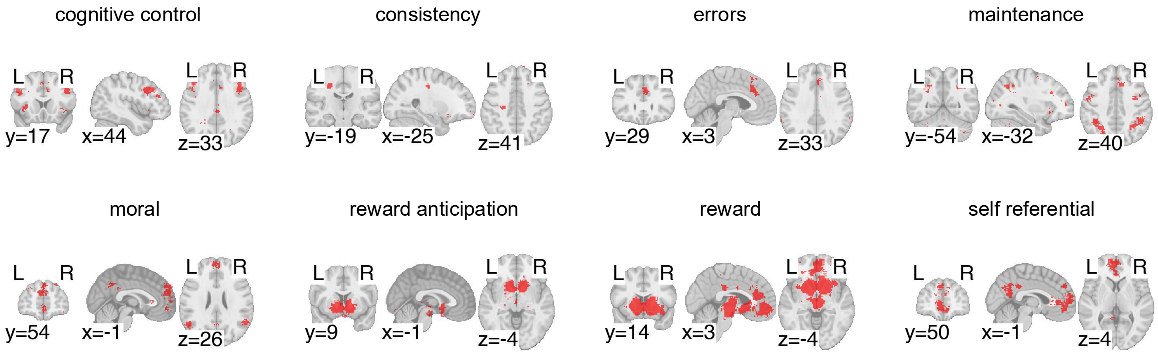

Fig. S2: **Neurosynth maps used for the neural pattern similarity analysis (using a criterion of a height threshold of** $p < 0.001$ **(z > 3.0))**

Supplementary results

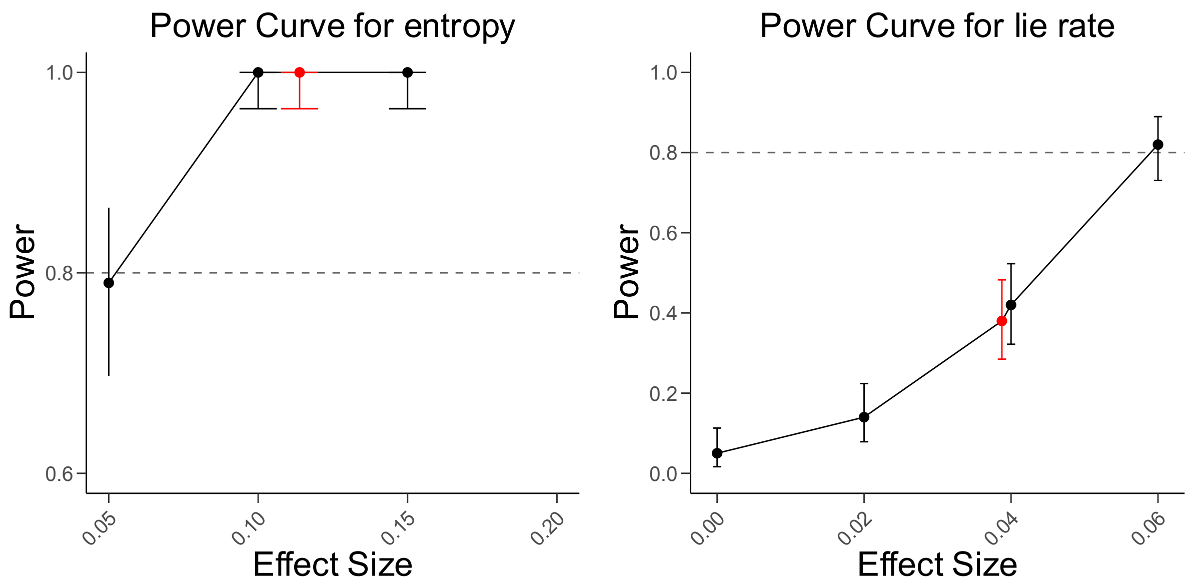

Fig. S3: **Sensitivity analysis results.** The results of the first LMM in the behavioral analysis (AUC $\sim$ lie rate + entropy + (1|sub_nr)).

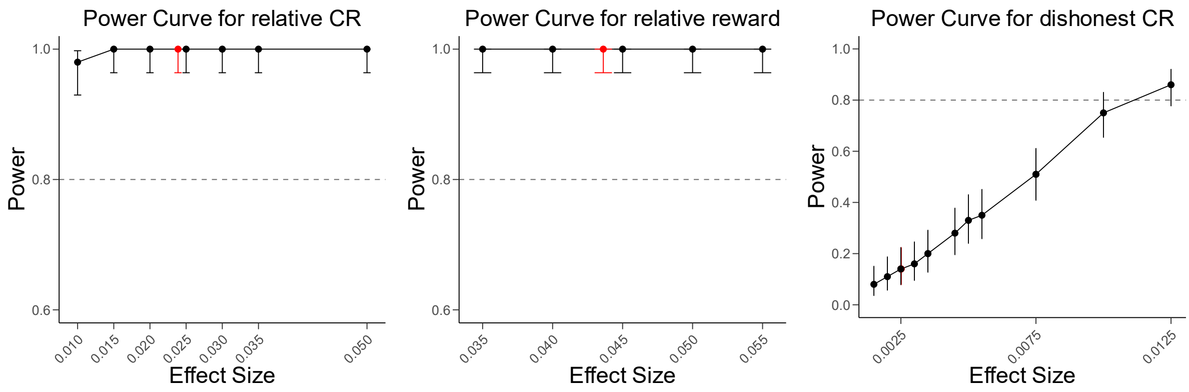

Fig. S4: **Sensitivity analysis results.** The results of the second LMM in the behavioral analysis (isLie $\sim$ dishonest CR + relative reward + relative CR + (1|subject)).

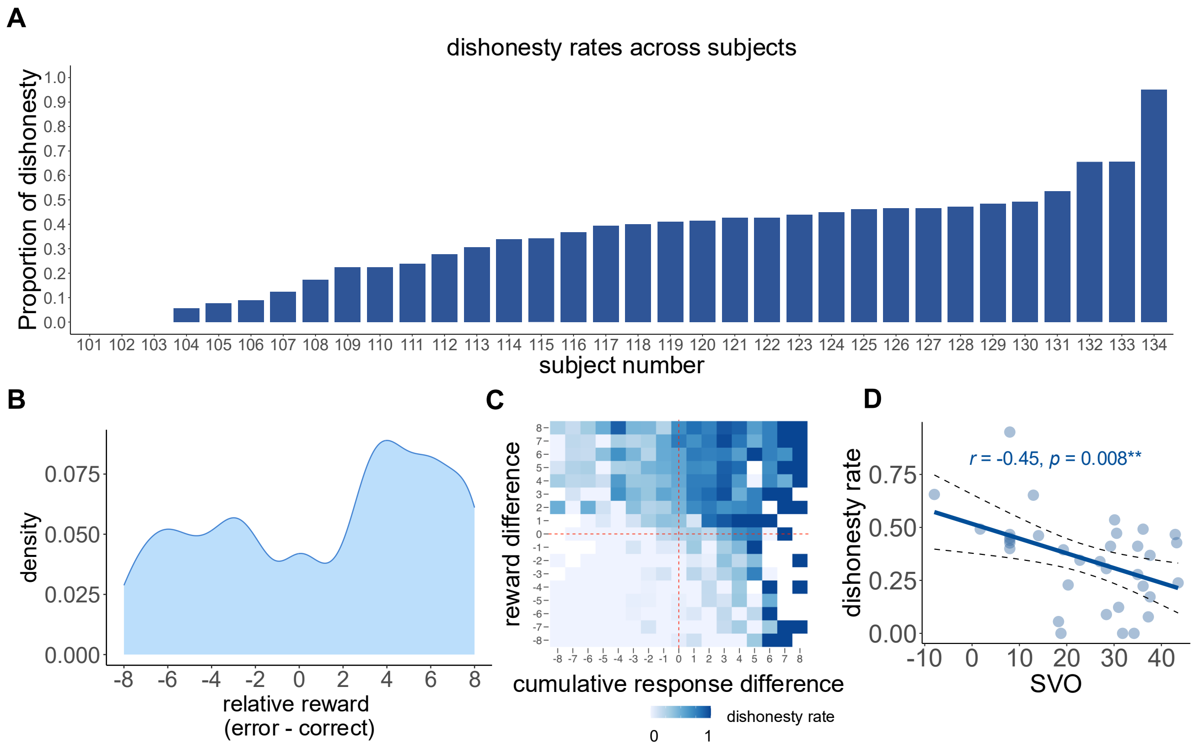

Fig. S5: **Supplementary behavioral results. (A)** Total lie rates across participants. We collected data from 34 participants where 3 subjects resisted the reward and didn't lie. **(B)** Density of reward difference between error and correct answers. In over 50% of the trials, the reward was higher for error choice to induce dishonesty. **(C)** lie rate as a function of relative reward and relative cumulative responses (CR). **(D)** Lie rate was negatively correlated with Social Value Orientation (SVO) score.

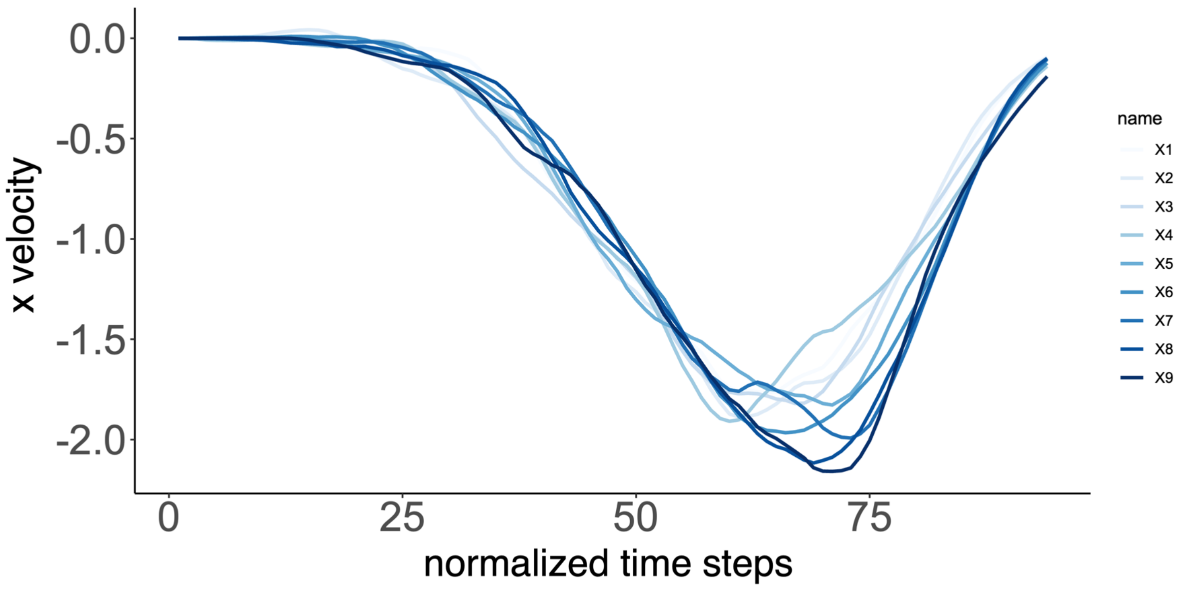

Fig. S6: **The velocity along the x direction of each run.** An increase in choice conflict was observed along with the session.

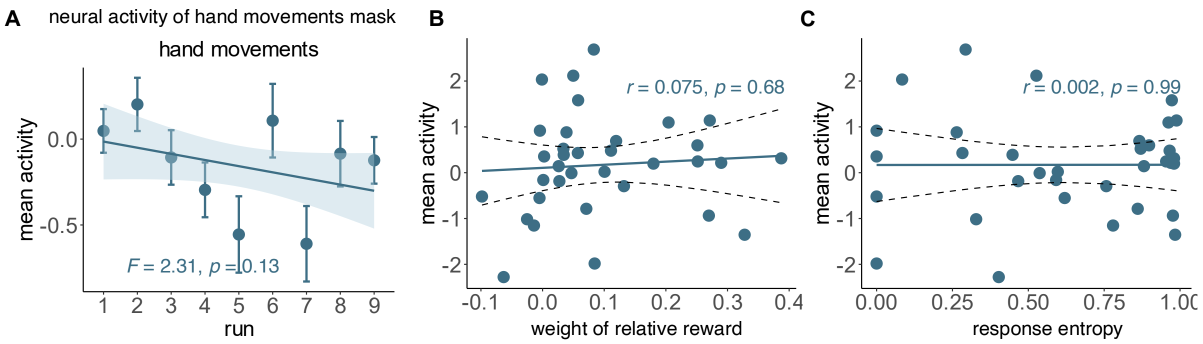

Fig. S7: **Results extracted from the hand movement mask. (A)** The activity of the hand movement mask didn't change across the runs ($F = 2.31$, $p = 0.13$, $\eta_p^2 = 0.008$). **(B)** The activity change from the first run to the last run had no correlation with the weight of relative reward ($r = 0.075$, $p = 0.68$, 95%CI from -0.27 to 0.40) and **(C)** response entropy ($r = 0.002$, $p = 0.99$, 95%CI from -0.34 to 0.34).

model	parameters	DIC
model 1	v ~ 1+ CR + reward<br>a ~ 1+ session<br>z ~ 1	15315
model 2	v ~ 1+ CR x session + reward<br>a ~ 1+ session<br>z ~ 1	15037
model 3	v ~ 1+ CR + reward x session<br>a ~ 1+ session<br>z ~ 1	15078
model 4	v ~ 1+ CR x session + reward x session<br>a ~ 1+ session<br>z ~ 1	14966
**model 5**	**v ~ 1+ CR x session + reward x session**<br>**a ~ 1+ session**<br>**z ~ 1+ session**	**14329**
model 6	v ~ 1+ CR x session + reward x session<br>a ~ 1+ session<br>z ~ 1+ session x history	14595
model 7	v ~ 1+ CR x session + reward x session + history<br>a ~ 1+ session<br>z ~ 1+ session x history	14352

Fig. S8: **Drift-diffusion model structure and comparison.** CR and reward represented modulation (or weight) of the relative reward and relative cumulative response on drift rate. '1' stands for a participant-specific constant (or intercept). a = decision threshold, z = initial bias, and v = drift rate. In all of these models, non-decision time (NDT) was not modulated by trial-by-trial parameters. DIC = deviance information criterion. from

stat	observed	predicted	std of predicted	credible	quantile	mahalanobis
accuracy	0.35	0.33	0.23	True	45.66	0.096
mean_ub	1.62	2.20	0.70	True	18.28	0.82
10q_ub	1.02	0.68	0.24	True	90.68	1.45
30q_ub	1.24	1.09	0.33	True	72.22	0.46
50q_ub	1.48	1.58	0.50	True	46.76	0.20
70q_ub	1.79	2.35	0.81	True	24.80	0.69
90q_ub	2.41	4.29	1.66	True	10.92	1.13
mean_lb	-1.58	-2.00	0.53	True	76.64	0.80
10q_lb	1.00	0.87	0.27	True	68.07	0.46
30q_lb	1.23	1.21	0.30	True	56.26	0.08
50q_lb	1.44	1.56	0.36	True	42.15	0.32
70q_lb	1.77	2.11	0.55	True	29.71	0.61
90q_lb	2.33	3.59	1.33	True	20.67	0.95

Table S2: **Posterior Predictive Checks for the preferred model (Model 5).** Notes: lb: lower bound; ub: upperbound; 10q $\sim$90q: 10th $\sim$90th quantile of RT distribution; Credible: whether observed data falls in the 95% credible interval of the simulated data; Mahalanobis: Mahalanobis distance of the observed data from the center of distribution of the simulated data.

Parameter	Gelman-rubin statistic
v_former_diff	1.00
v_ses	1.01
v_former_diff:ses	1.00
v_diff	1.00
v_diff:ses	1.03
a_ses	1.01
z_ses	1.00

Table S3: **Convergence statistics for the model parameters of the preferred model (model 5).** Values close to 1 indicated successful convergence of the Markov-Chain Monte-Carlo sampler.

References

Avants, B., Epstein, C., Grossman, M., & Gee, J. (2008). Symmetric diffeomorphic image registration with cross-correlation: Evaluating automated labeling of elderly and neurodegenerative brain. *Medical Image Analysis*, *12*(1), 26-41. Retrieved from `http://www.sciencedirect.com/science/article/pii/S1361841507000606` doi: doi:10.1016/j.media.2007.06.004

Behzadi, Y., Restom, K., Liau, J., & Liu, T. T. (2007). A component based noise correction method (CompCor) for BOLD and perfusion based fmri. *NeuroImage*, *37*(1), 90-101. Retrieved from `http://www.sciencedirect.com/science/article/pii/S1053811907003837` doi: doi:10.1016/j.neuroimage.2007.04.042

Cox, R. W., & Hyde, J. S. (1997). Software tools for analysis and visualization of fmri data. *NMR in Biomedicine*, *10*(4-5), 171-178. doi: doi:10.1002/(SICI)1099-1492(199706/08)10:4/5<171::AID-NBM453>3.0.CO;2-L

Esteban, O., Markiewicz, C. J., Blair, R. W., Moodie, C. A., Isik, A. I., Erramuzpe, A., … others (2019). fmriprep: a robust preprocessing pipeline for functional mri. *Nature methods*, *16*(1), 111–116. doi: doi:10.1038/s41592-018-0235-4

Fonov, V., Evans, A., McKinstry, R., Almli, C., & Collins, D. (2009). Unbiased nonlinear average age-appropriate brain templates from birth to adulthood. *NeuroImage*, *47, Supplement 1*, S102. doi: doi:10.1016/S1053-8119(09)70884-5

Gorgolewski, K., Burns, C. D., Madison, C., Clark, D., Halchenko, Y. O., Waskom, M. L., & Ghosh, S. S. (2011). Nipype: a flexible, lightweight and extensible neuroimag-

ing data processing framework in python. *Frontiers in neuroinformatics*, *5*, 13. doi: doi:10.3389/fninf.2011.00013

Greve, D. N., & Fischl, B. (2009). Accurate and robust brain image alignment using boundary-based registration. *NeuroImage*, *48*(1), 63-72. doi: doi:10.1016/j.neuroimage.2009.06.060

Jenkinson, M., Bannister, P., Brady, M., & Smith, S. (2002). Improved optimization for the robust and accurate linear registration and motion correction of brain images. *NeuroImage*, *17*(2), 825-841. Retrieved from `http://www.sciencedirect.com/science/article/pii/S1053811902911328` doi: doi:10.1006/nimg.2002.1132

Jenkinson, M., & Smith, S. (2001). A global optimisation method for robust affine registration of brain images. *Medical Image Analysis*, *5*(2), 143–156. Retrieved 2018-07-27, from `http://www.sciencedirect.com/science/article/pii/S1361841501000366` doi: doi:10.1016/S1361-8415(01)00036-6

Lanczos, C. (1964). Evaluation of noisy data. *Journal of the Society for Industrial and Applied Mathematics Series B Numerical Analysis*, *1*(1), 76-85. Retrieved from `http://epubs.siam.org/doi/10.1137/0701007` doi: doi:10.1137/0701007

Power, J. D., Mitra, A., Laumann, T. O., Snyder, A. Z., Schlaggar, B. L., & Petersen, S. E. (2014). Methods to detect, characterize, and remove motion artifact in resting state fmri. *NeuroImage*, *84*(Supplement C), 320-341. Retrieved from `http://www.sciencedirect.com/science/article/pii/S1053811913009117` doi: doi:10.1016/j.neuroimage.2013.08.048

Satterthwaite, T. D., Elliott, M. A., Gerraty, R. T., Ruparel, K., Loughead, J., Calkins, M. E., … Wolf, D. H. (2013). An improved framework for confound regression and filtering for control of motion artifact in the preprocessing of resting-state functional connectivity data. *NeuroImage*, *64*(1), 240–256. Retrieved from `http://linkinghub.elsevier.com/retrieve/pii/S1053811912008609` doi: doi:10.1016/j.neuroimage.2012.08.052

Tustison, N. J., Avants, B. B., Cook, P. A., Zheng, Y., Egan, A., Yushkevich, P. A., & Gee, J. C. (2010). N4itk: Improved n3 bias correction. *IEEE Transactions on Medical Imaging*, *29*(6), 1310-1320. doi: doi:10.1109/TMI.2010.2046908

Zhang, Y., Brady, M., & Smith, S. (2001). Segmentation of brain MR images through a hidden markov random field model and the expectation-maximization algorithm. *IEEE Transactions on Medical Imaging*, *20*(1), 45-57. doi: doi:10.1109/42.906424
